# Molecular basis for the distinct functions of redox-active and FeS-transfering glutaredoxins

**DOI:** 10.1038/s41467-020-17323-0

**Published:** 2020-07-10

**Authors:** Daniel Trnka, Anna D. Engelke, Manuela Gellert, Anna Moseler, Md Faruq Hossain, Tobias T. Lindenberg, Luca Pedroletti, Benjamin Odermatt, João V. de Souza, Agnieszka K. Bronowska, Tobias P. Dick, Uli Mühlenhoff, Andreas J. Meyer, Carsten Berndt, Christopher Horst Lillig

**Affiliations:** 1 Institute for Medical Biochemistry and Molecular Biology, University Medicine, University of Greifswald, Greifswald, Germany; 2 Department of Neurology, Medical Faculty, Heinrich-Heine University Düsseldorf, Düsseldorf, Germany; 3 Institute of Crop Science and Resource Conservation, University of Bonn, Bonn, Germany; 4 Institute of Neuroanatomy, University Clinics, University of Bonn, Bonn, Germany; 5 Chemistry, School of Natural and Environmental Sciences, Newcastle University, Newcastle, NE1 7RU UK; 6 Division of Redox Regulation, DKFZ-ZMBH Alliance, German Cancer Research Center (DKFZ), Heidelberg, Germany; 7 Institute for Cytobiology and Cytopathology, Philipps University Marburg, Marburg, Germany; 8 Present Address: UMR 1136 Interactions Arbres/Microorganismes, Université de Lorraine, Vandoeuvre-lès-Nancy, France

**Keywords:** Biocatalysis, Enzyme mechanisms, Cell signalling, Iron, Protein design

## Abstract

Despite their very close structural similarity, CxxC/S-type (class I) glutaredoxins (Grxs) act as oxidoreductases, while CGFS-type (class II) Grxs act as FeS cluster transferases. Here we show that the key determinant of Grx function is a distinct loop structure adjacent to the active site. Engineering of a CxxC/S-type Grx with a CGFS-type loop switched its function from oxidoreductase to FeS transferase. Engineering of a CGFS-type Grx with a CxxC/S-type loop abolished FeS transferase activity and activated the oxidative half reaction of the oxidoreductase. The reductive half-reaction, requiring the interaction with a second GSH molecule, was enabled by switching additional residues in the active site. We explain how subtle structural differences, mostly depending on the structure of one particular loop, act in concert to determine Grx function.

## Abstract (web summary)

Glutaredoxins are a family of essential enzymes divided into two major classes with either a CGFS or a CxxC active site, of which only the latter exhibits oxidoreductase activity. Here the authors address the structural basis for the functional difference between the two classes of glutaredoxins.

## Introduction

Glutaredoxins (Grxs) were first described as electron donors for essential metabolic processes, such as the reduction of ribonucleotides and activated sulfate^1–3^. These initially described Grxs (also known as class I or dithiol Grxs), herein termed CxxC/S-type Grxs, share a consensus CPYC active site motif that enables the proteins to catalyze the glutathione (GSH)-dependent reduction of protein disulfides. Later on, these Grxs were also recognized for their ability to specifically reduce mixed disulfides formed between protein thiols and GSH, a reaction termed de-glutathionylation^4^. A subgroup of the CxxC/S-type Grxs in which the prolyl residue in the active site is replaced by seryl or glycyl residues (C(S/G)YC) can complex a Fe_2_S_2_ cluster at the interface of a dimeric complex of two Grxs ligated by the two N-terminal active site thiols and the thiols of two non-covalently bound GSH molecules^5–9^.The de-glutathionylation reaction requires only the more N-terminal cysteinyl residue in the active site of the CxxC/S-type Grxs and was thus termed the monothiol reaction mechanism. In brief, the reaction is initiated by a nucleophilic attack of the thiolate of this cysteinyl residue on the glutathione moiety of the glutathionylated protein. This results in the reduction of the protein substrate and a mixed disulfide between the N-terminal cysteinyl residue of the Grx active site and GSH (Grx-S-SG). Subsequently, this disulfide is reduced by a second molecule of GSH. The basis for this reduction of the mixed disulfide is a second GSH binding site on the Grxs that was suggested to activate GSH as the reducing agent^10^. In general, most CxxC/S-type Grxs also catalyze the direct reduction of protein disulfides in a thiol^−^disulfide exchange reaction (termed dithiol reaction mechanism, summarized, e.g., in ref. ^11^). In some cases, for instance the reduction of mammalian ribonucleotide reductase by Grx1, protein disulfides may react with GSH first yielding a glutathionylated protein that is subsequently reduced by the Grx in the monothiol reaction mechanism^12^. It is important to state that all these reactions and steps are reversible equilibrium reactions, the direction of which is defined by thermodynamic constrains, i.e. the concentrations and redox potential of the GSH/glutathione disulfide (GSSG) redox couple. Under conditions that lead to a transient increase in the amounts of GSSG, Grxs will catalyze the glutathionylation of proteins at the expense of GSSG. In fact, the oxidation of the engineered redox-sensitive roGFP2 disulfide by GSSG was suggested to be catalyzed in the monothiol reaction mechanism^13^.

Around 30 years following the first description of the CxxC/S-type Grxs, a second group of Grxs came into focus. These proteins share a consensus CGFS active site motif and are also known as class II or monothiol Grxs, herein termed CGFS-type Grxs. These proteins function in iron metabolism, i.e. bacterial, mitochondrial, or plastidal FeS cluster biogenesis^14–19^ and the trafficking of iron in the cytosol of eukaryotic cells^20,21^. These functions critically depend on the ability of the CGFS-type Grxs to bind a Fe_2_S_2_ cluster in a mode similar to those of the CxxC/S-type Grxs^22,23^. Still, the function of the CGFS-type Grxs could not be rescued by FeS-coordinating CxxC/S-type Grxs in a yeast Δ*grx5* mutant, indicating different roles of the coordinated clusters for the functions of both classes of Grxs^15^. Both Grx classes share the same basic fold, including all motifs required for the interaction with GSH moieties^24^. The more N-terminal active site cysteinyl residue is fully conserved between all classes of Grxs. Despite of the presence of all these features required for the activity as GSH-dependent oxidoreductase, CGFS-type Grxs lack significant activity with established model substrates or physiological substrates of the CxxC/S-type Grxs (summarized, e.g., in ref. ^25^).

In this study, we re-investigated the structural differences of the two main classes of Grxs to solve the mystery of the missing FeS transferase activity of the CxxC/S-type and the lack of oxidoreductase activity of the CGFS-type Grxs. We hypothesized that not a radically different substrate specificity accounts for the lack of activity, but rather slightly different modes of GSH binding. The validity of our hypothesis was analyzed in vitro and in vivo using engineered mutants from both Grx subfamilies.

## Results

### Major differences in substrate specificity are unlikely

One proposed explanation for the lack of significant oxidoreductase activity of the CGFS-type Grxs may be that their substrate specificity differs radically from those of the CxxC/S-type Grxs. Substrate recognition and interaction of the Grxs is largely determined by their electrostatic properties^26^. We have compared the electrostatic properties of both the types of Grxs from various species (Supplementary Fig. 1). These demonstrate a considerable degree of similarity between the surface potential at and surrounding the active sites. These features, together with the conservation of all four motifs required for GSH binding^24^, do not support a radically different substrate specificity or reaction mechanisms as the reason for the lack in oxidoreductase activity of the CGFS-type Grxs.

### Alternative loop structures are the main difference

**Fig. 1:**
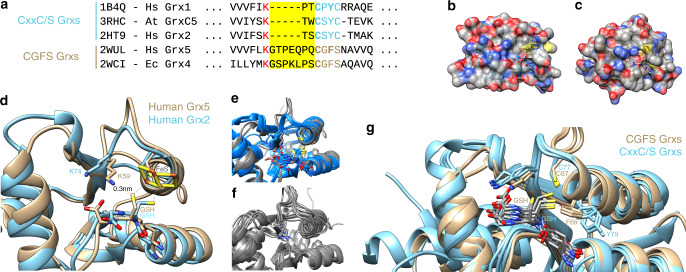
Structural analysis of CxxC/S- and CGFS-type glutaredoxins. **a** Primary structure comparison of glutaredoxins from both classes around the active site: CxxC/S-type: Homo sapiens (Hs) Grx1 (gene glrx1, PDB 1B4Q), *Arabidopsis thaliana* (At) GrxC5 (gene GrxC5, PDB 3RHB), and Hs Grx2 (GLRX2, PDB 2HT9). CGFS-type: Hs Grx5 (GLRX5, PDB 2WUL) and *Escherichia coli* (Ec) Grx4 (grxD, PDB 1YKA). The alignment was generated by super-positioning of the 3D structures of the PDB entries. The critical lysyl residue is highlighted in red, the yellow box the class-specific loops, the active sites are shown in blue and brown according to the class. **b** Surface representation of one monomer of the human Grx2 holo-complex with bound GSH and Fe_2_S_2_ cluster (PDB 2HT9). **c** Surface representation of one monomer of the human Grx5 holo-complex with bound GSH and Fe_2_S_2_ cluster (PDB 2WUL). **d** Super-positioning of the structures of the CxxC/S-type human Grx2 (blue) and the CGFS-type human Grx5 (brown) including their bound FeS clusters and glutathione. **e** Comparison of the structures of all CxxC/S-type (blue) and CGFS-type (gray) Grxs with non-covalently bound GSH (PDB entries used: CGFS-type Grxs, 2XCI, 2WUL, 3RHC, and 5J3R; CxxC/S-type Grxs, 1B4Q, 2E7P, and 2HT9). **f** Comparison of the critical lysyl residue in all structures of CGFS-type Grxs (PDB entries: 2LKU, 2MNZ, 2WCI, 2WUL, 2YAN, 3GX8, 2IPZ, and 2ZYW). **g** Position of the phenylalanyl and tyrosyl residues in CGFS- (brown) and CxxC/S-type (blue) Grxs in relation to the GSH thiol (PDB entries used: CGFS-type Grxs, 2WUL and 3RHC; CxxC/S-type Grxs, 1B4Q, 2E7P, and 2HT9.

Next, we re-evaluated the structural differences between two Grx classes in more detail (Fig. 1). The general fold in both classes of Grxs is very similar following the classical thioredoxin fold^27^. This is also true for the binding of GSH. Figure 1b, c depict GSH bound to the surfaces of human Grx2 (CxxC/S-type, b) and human Grx5 (CGFS-type, c) from the structures of the Fe_2_S_2_ holo-complexes deposited in the PDB. As discussed earlier^10,24^, both classes use the same four motifs to bind GSH non-covalently for the ligation of the FeS cluster and redox reactions, respectively. The largest deviation between the substrate binding sites of both Grxs was identified in the loop region directly adjacent to the more N-terminal active site cysteinyl residue. All CGFS-type Grxs contain an extension in this loop of 5 amino acid residues (Fig. 1a, yellow box). The N-terminal anchor point of this loop is a lysyl residue (Fig. 1a, red). This lysyl or (very rarely) an alternative arginyl residue is strictly conserved in all Grxs. In CxxC/S-type Grxs, the positive charge electrostatically interacts with the carboxyl group of the C-terminal glycyl residue of the GSH molecule. In the CGFS-type Grxs, however, the conformation of the extended loop following this residue shifts the orientation of the ε-amino group towards the thiol of the GSH molecule by 0.2 nm. This causes a re-orientation of the GSH thiol towards the amino group (Fig. 1d, e); in the CxxC/S-type Grxs this distance is >0.88 nm. This structural difference is not restricted to the two human mitochondrial Grxs. In fact, all deposited structures of CGFS-type and CxxC/S-type Grxs with non-covalently bound GSH show the same distinct features (Fig. 1e, blue: CxxC/S-type Grxs, gray: CGFS-type Grxs). The re-orientation of the lysine side chain is not dependent on binding of GSH, it is a feature present in all experimentally solved structures of the CGFS-type Grxs (Fig. 1f). Another conserved difference between the structures of the two classes is the orientation of the active site phenylalanyl and tyrosyl residues in the active sites, respectively (Fig. 1g). The position of the Phe side chain in the CGFS-type Grxs would, in fact, clash with the thiol of the GSH as bound in the CxxC/S-type Grxs. It will thus contribute to the different orientation of the GSH thiol groups in both classes of Grxs. These different orientations do profoundly affect the ligation and orientation of the Fe_2_S_2_ clusters and thus the overall conformation of the holo-complexes. Moreover, they will also affect the reactivity of the GSH thiol, and thus the formation of the mixed disulfide with the N-terminal active site thiol of the Grx. The alternative orientations of the active site phenylalanyl versus tyrosyl residues, together with the different orientation of the GSH thiols, will also affect the attack of the second GSH on a Grx-GSH mixed disulfide intermediate, as this has to occur in an angle of 180°^28^.

**Fig. 2:**
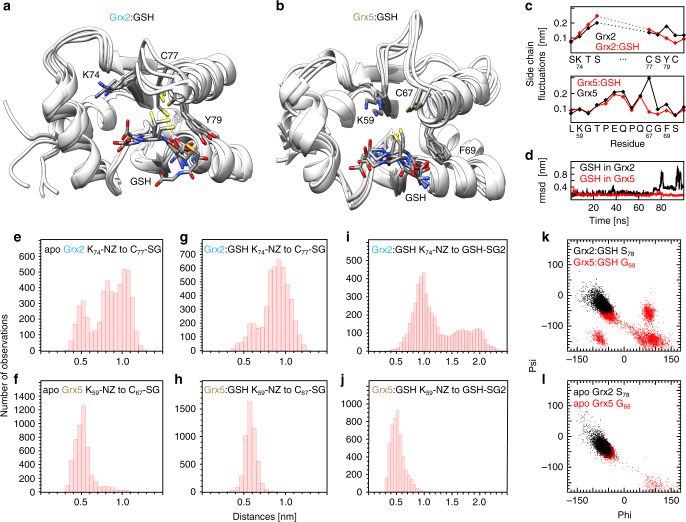
Molecular dynamics simulations of human Grx2 and Grx5 GSH complexes. **a**, **b** The four most representative structures of the simulations runs of the Grx2:GSH (**a**) and the Grx5:GSH (**b**) complexes. The structures were identified with the UCSF chimera ‘Ensemble Cluster tool’ and represent 76% (Grx2:GSH) and 65% (Grx5:Grx) of all structures. **c** Side chain fluctuations of the residues of the loops and active sides in the Grx2:GSH complex and the Grx5:GSH complex, black squares: apo Grxs, red squares: Grx:GSH complexes. **d** Root mean square deviation (rmsd) of the GSH molecule bound in the complexes, black line: Grx2, red line: Grx5. **e**–**j** Distribution of distances of the indicated atom pairs over three independent simulations of 100 ns each. **e**–**h** Distances of the amino nitrogen atom of the strictly conserved lysyl residue to the thiol sulfur of the more N-terminal active site cysteinyl residue in apo Grx2 (**e**), apo Grx5 (**f**), the Grx2:GSH complex (**g**), and the Grx5:GSH complex (**h**). **i**, **j** Distances between the amino nitrogen atom of the strictly conserved lysyl residue to the thiol sulfur of the non-covalently bound GSH in the Grx2:GSH complex (**e**) and the Grx5:GSH complex (**f**). **k**–**l** Distribution of phi and psi angles of the active site seryl residue in Grx2 (black) and the respective glycyl residue in Grx5 (red) in the Ramachandran plots for the Grx:GSH complexes (**k**) and the apo proteins (**l**).

We performed all-atom molecular dynamics (MD) simulations with Grx2 and Grx5 as apo proteins and non-covalent Grx:GSH complexes for 100 ns. The results obtained confirm the distinct orientations of the GSH thiols and the conserved lysyl residues in both classes of Grxs (Fig. 2). Moreover, the simulations also indicate that the alternative orientation of the lysyl amino group is independent of GSH binding. The distance of the amino nitrogen of the lysyl residue in Grx2 to the more N-terminal active site thiol is either around 0.5 nm or around 0.8 nm (Fig. 2e), the larger distance is stabilized in the Grx2:GSH complex (Fig. 2a, g). For Grx5 it is stable around 0.5 nm independent of GSH binding (Fig. 2b, f, h). As seen in the crystal and NMR structures (Fig. 1d–f), the distance of the amino nitrogen to the GSH thiol is significantly larger in Grx2 (up to 2 nm, Fig. 2a, i) than in Grx5 (around 0.5 nm, Fig. 2b, j). The different orientations of the Phe versus Tyr residues in the active sites may be the result of the glycyl residue in the CGFS active site. This allows for alternative backbone conformations and favors slightly shifted phi-psi bond angles compared to the seryl residue in Grx2 (Fig. 2k, l). Noteworthy, in Grx5, but not Grx2, the orientation of the N-terminal active side thiol is locked in the presence of GSH (Fig. 2c). Our simulations also indicate that Grx5 binds GSH with higher binding energy, in fact the non-covalent Grx2:GSH complex partially dissociated during the simulation (Fig. 2d).

Based on our structural analyses, we proposed that alternative loop and active site structures are the molecular basis for the lack of activities of the CGFS-type Grxs as oxidoreductases and the CxxC/S-type Grxs as FeS transferases.

### Mutants mimicking the alternative loop structures

To test our hypothesis, we have generated mutants of both human Grx2 and Grx5 (Supplementary Table 1). First, we have inserted the five amino acid extension GTPEQ into the structure of the CxxC/S-type Grx2 (Grx2-loop; Grx2 with a CGFS-type loop). Secondly, we shortened the loop in the CGFS-type Grx5 to enforce a CxxC/S-type conformation of the lysl side chain (Grx5-loop; Grx5 with a CxxC/S-type loop). In addition, we exchanged the CGFS active site of Grx5 to the Grx2 CSYC motif in both wild-type (Grx5-AS) and CxxC/S-type loop modified Grx5 (Grx5-loop/AS). All proteins were expressed recombinantly and purified as His-tagged proteins. Their structural stability was confirmed by differential scanning fluorimetry (thermofluor assay, Supplementary Fig. 2). All proteins were found to be thermally stable at the designated assay temperatures of 25 °C (activity assays) and 28 °C (zebrafish), the *T*_m_ for their denaturation was in between 35 °C and 57 °C.

### Redox activity of the engineered proteins

**Fig. 3:**
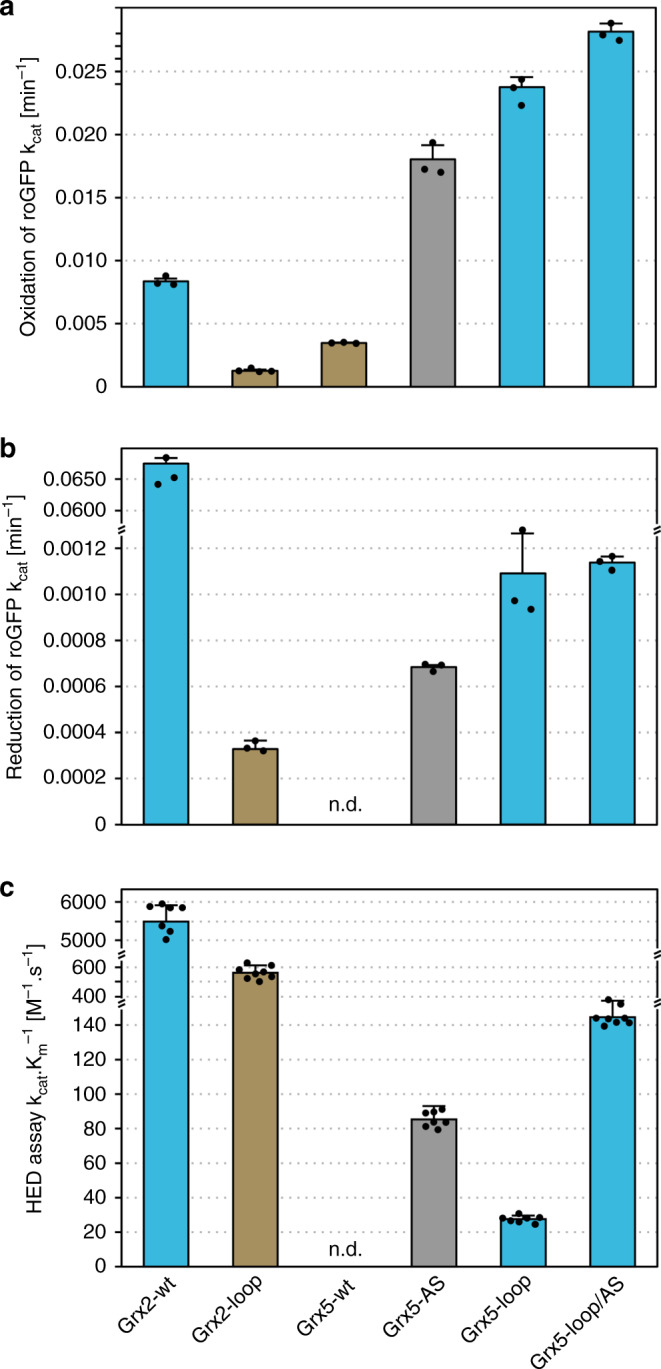
Comparison of the catalytic activities of the wild-type and engineered Grxs. **a** Catalytic activity (*k*_cat_) of the Grxs in the oxidation of roGFP2. **b** catalytic activity (*k*_cat_) of the Grxs in the reduction of roGFP2; *k*_cat_ here was defined as the specific initial rate of roGFP oxidation and reduction, respectively, at 1µmol l^−1^ substrate concentration. **c** Catalytic efficiency (*k*_cat_·*K*_m_^−1^) of the Grxs in the HED assay. All data are shown as mean ± sd. The roGFP2 assays were all performed at four different Grx concentrations with three replicates each, the HED assay data represent the mean of *n* = 7–8. n.d. not detected. Blue bars represents the proteins with the CxxC/S-type loop, brown bars proteins with the CGFS-type loop, the gray bar Grx5 with the CSYC active site of Grx2. Source data are provided as a Source data file.

We analyzed the oxidoreductase activity of the wild-type and mutant proteins using three different assays (Table 1, Fig. 3). The redox sensitive roGFP2^29^ allows to follow both the oxidation and the reduction of a target protein. The reaction is thought to occur in three reversible steps through the glutathionylation of the protein before the formation of an intra-molecular disulfide:

1 $${\mathrm{Grx}} - {\mathrm{SH}} + {\mathrm{GSSG}} \rightleftharpoons {\mathrm{Grx}} - {\mathrm{S}} - {\mathrm{SG}} + {\mathrm{GSH}}$$

2 $${\mathrm{Grx}} - {\mathrm{S}} - {\mathrm{SG}} + {\mathrm{roGFP}}2 - \left( {{\mathrm{SH}}} \right)_2 \rightleftharpoons {\mathrm{Grx}} - {\mathrm{SH}} + {\mathrm{roGFP}}2 - {\mathrm{S}} - {\mathrm{SG}}$$

3 $${\mathrm{roGFP}}2 - {\mathrm{S}} - {\mathrm{SG}} \rightleftharpoons {\mathrm{roGFP}}2 - \left( {{\mathrm{S}} - {\mathrm{S}}} \right) + {\mathrm{GSH}}$$

**Table 1:** Kinetic data of the proteins analyzed.

Protein	HED assay			roGFP2 reduction	roGFP2 oxidation
	*K*_*m* app_ (HED)	*k*_cat_	*k*_cat_·*K*_*m*_^−1^	*k*_cat_	*k*_cat_
	µM	s^−1^	M^−1^ s^−1^	min^−1^	min^−1^
AtGrxC1	773 ± 164	4.25 ± 0.27	5.50 × 103	(1.80 ± 0.28) × 10^−1^	(4.49 ± 0.23) × 10^−2^
Grx2-wt	165 ± 12	0.91 ± 0.03	5.54 × 103	(6.55 ± 0.17) × 10^−2^	(8.35 ± 0.26) × 10^−3^
Grx2-loop	317 ± 32	0.180 ± 0.01	5.68 × 102	(3.38 ± 0.24) × 10^−4^	(2.11 ± 0.11) × 10^−3^
Grx5-wt	–	0	–	0	(3.50 ± 0.07) × 10^−3^
Grx5-AS	771 ± 64	0.07 ± 0.00	8.50 × 101	(6.68 ± 0.09) × 10^−4^	(1.79 ± 0.13) × 10^−2^
Grx5-loop	818 ± 51	0.02 ± 0.00	2.80 × 101	(1.09 ± 0.23) × 10^−3^	(2.38 ± 0.08) × 10^−2^
Grx5-loop/AS	924 ± 54	0.13 ± 0.00	1.45 × 102	(1.14 ± 0.02) × 10^−3^	(2.81 ± 0.06) × 10^−2^

All data are shown as mean ± sd (*n* = 7–8 for the HED assay, *n* = 4 (with 3 technical replicates) for the roGFP2 assays. Source data are provided as a Source data file.

We have confirmed this reaction sequence using mass spectrometry and a roGFP2 mutant lacking the second cysteinyl residue required for the reaction (Eq. 2) to trap the glutahionylated roGFP2 intermediate (summarized in Supplementary Fig. 3). Notably, this roGFP2-SG intermediate is spectroscopically indistinguishable from the reduced protein. The kinetics of fluorescence changes must therefore reflect the overall reaction. In the reaction of the Grx-catalyzed oxidation of the 2-Cys roGFP2 by GSSG no significant amounts of the roGFP2-SG intermediate could be detected. We feel thus confident to conclude that oxidation of roGFP2 through Grxs is facilitated in steps 1–2–3 (Eqs. 1–3); reaction (2) is rate limiting for the full oxidation of roGFP2 and the ratiometric change in fluorescence excitation.

Grx2 catalyzed the reaction at reasonable rates, comparable to those of the classical CxxC-type Arabidopsis thaliana GrxC1 that was used as a highly efficient positive control in all reactions (Table 1, Supplementary Figs. 4 and 5). Introduction of the alternative loop in the Grx2-loop mutant led to a loss of 75% of its activity in the oxidative reaction. This reaction was also the only one in which wild-type Grx5 (Grx5-wt) showed some activity, ~42% of the activity of Grx2. Exchange of the active site to those of Grx2 increased the activity to 214%. The introduction of the CxxC/S-type loop increased the activity further to 285%, the combined Grx5-loop/AS mutation to 337% (Fig. 3a, details in Supplementary Fig. 4). Clearly, the shortening of the loop alone was sufficient to turn Grx5 into a highly efficient catalyst of roGFP2 oxidation.

Reduction of oxidized roGFP2 takes place in reaction order 3–2–1 (Eqs. 1–3, see above). The extension of the loop in the Grx2-loop mutant decreased the rate to 0.5%. Grx5-wt is inactive in this reaction, exchange of the active site (Grx5-AS) yielded an activity of ~1.1% of Grx2-wt. The CxxC/S-type loop in the Grx5-loop mutant increased the rate to 1.7% compared to Grx2-wt with some additional additive effect of the active site swap (Table 1, Fig. 3b, Supplementary Fig. 5). The shortening of the loop in the Grx5-loop mutant allowed the protein to facilitate the reaction, albeit at relatively low rates. Introduction of the extended CGFS-type loop diminished the oxidoreductase function of Grx2 with roGFP2 as model substrate.

The standard assay for Grxs is the so called HED (hydoxyethyl disulfide) assay. HED is a disulfide between two β-mercapto ethanol (βME) molecules. This assay requires both oxidative (5) and reductive (6) half reactions:

4 $${\mathrm{HED}} + {\mathrm{GSH}} \rightleftharpoons \beta {\mathrm{ME}} - {\mathrm{SG}} + {\mathrm{GSH}}\left( {{\mathrm{non}}\,{\mathrm{enzymatically}}} \right)$$

5 $$\beta {\mathrm{ME}} - {\mathrm{SG}} + {\mathrm{Grx}} - {\mathrm{SH}} \rightleftharpoons \beta {\mathrm{ME}} + {\mathrm{Grx}} - {\mathrm{S}} - {\mathrm{SG}}$$

6 $${\mathrm{Grx}} - {\mathrm{S}} - {\mathrm{SG}} + {\mathrm{GSH}} \rightleftharpoons {\mathrm{Grx}} - {\mathrm{SH}} + {\mathrm{GSSG}}$$

The HED assay is numerically not exact, since [HED] in the assay does not correspond to the [βME] in a 1:1 stoichiometric manner^30^. The assay does however allow to compare apparent catalytic efficiencies. Grx2 with the extended CGFS-type loop lost 90% of the wild-type protein’s catalytic efficiency (Table 1, Fig. 3c, Supplementary Fig. 6). No activity could be recorded for Grx5-wt in this assay. All engineered variants, however, displayed clear activity: Grx5-AS at 1.5% of Grx2-wt, Grx5-loop at 0.5% and the combined Grx5-loop/AS protein at 2.6% (Fig. 3c).

We hypothesized that this markedly increased activity in the oxidation of roGFP2 resulted from an increased reactivity of the protein with glutathione disulfide moieties yielding the Grx-S-SG mixed disulfide (reaction 1). Oxidation of reduced Grx5-wt with GSSG led to 0.53 ± 0.10 mixed disulfides per monomer in equilibrium, the reaction of Grx5-loop with GSSG to 0.91 ± 0.10 Grx-S-SG mixed disulfides (Supplementary Fig. 7).

The oxidation and reduction of roGFP2 requires the formation of an intermediate protein-glutahione mixed disulfide (see above). To analyze the ability of the Grxs to facilitate the reversible (de)-glutathionylation of other proteins as well, we have analyzed their ability to de-glutathionylate proteins in HeLa cell extracts, and purified BSA and Sirt1 (Supplementary Fig. 8). In all cases, the Grx5 double mutant Grx5-loop/AS de-glutathionylated the proteins most efficiently, while—with the exception of Sirt1—both the Grx5-loop and Grx5-AS mutants were less efficient. Wild-type Grx5 showed low (HeLa extract and BSA) or no (Sirt1) activity.

### Effect of the mutations on FeS cluster binding and stability

Our structural analysis revealed significant differences in the orientation of the GSH thiol in both Grx classes. This thiol is also one of the ligands for the Fe_2_S_2_ cluster bound to the dimeric holo-complex of the proteins (Fig. 1). All wild-type and mutant proteins were isolated as brownish FeS proteins from *E. coli*. To ensure similar FeS occupancy of all proteins, we reconstituted the Fe_2_S_2_ cluster in both wild-type and mutant Grx2 and Grx5, respectively. We have quantified the FeS content of the reconstituted proteins from molar absorptivity and by colorimetric methods. The results, summarized in Supplementary Table 2, demonstrate a similar FeS occupancy for all proteins at around 70%.

**Fig. 4:**
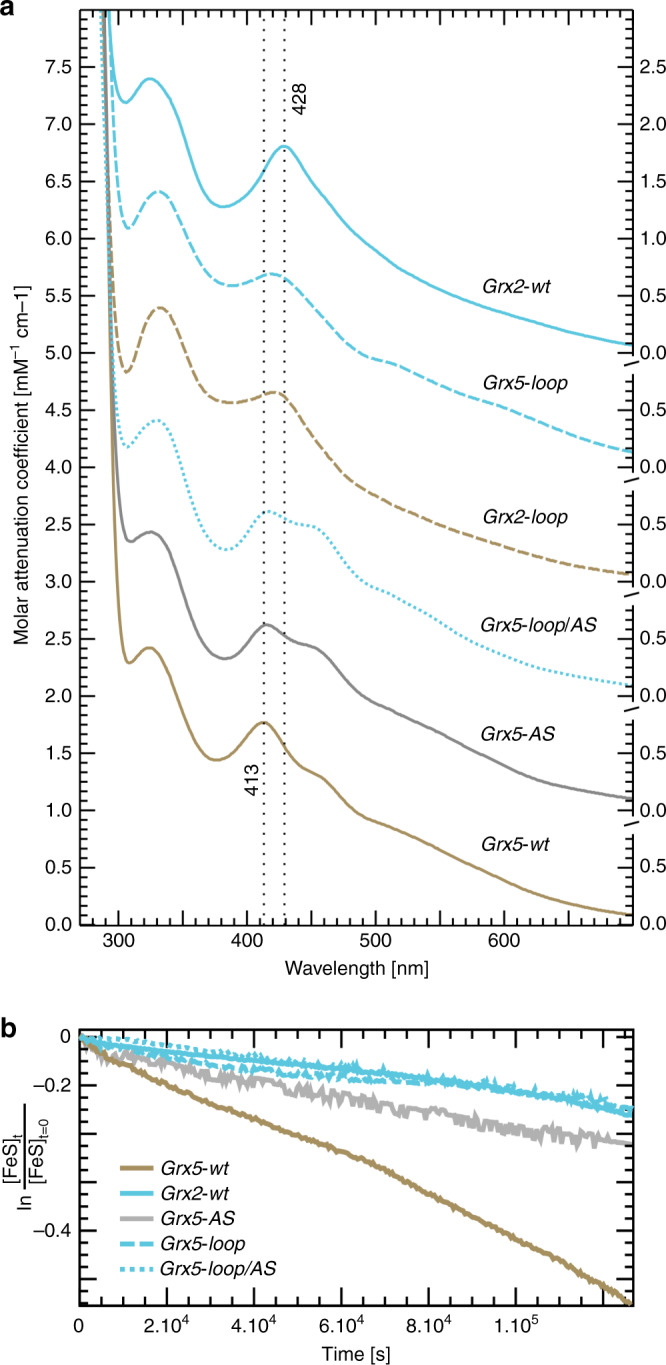
FeS cluster binding of the wild-type and mutant Grx2 and Grx5. **a** Spectra of in vitro reconstituted holo-complexes as indicated. The upper spectra were all shifted 1 unit of mM^−1^ cm^−1^ upwards for clarity. The dotted vertical lines indicate the calculated maximum of the absorption peak of wild-type Grx2 and Grx5, respectively. The spectra were normalized to the molar absorptivity of the proteins calculated from their primary structures using ProtParam. **b** Integrated first order kinetics of the decay of the FeS clusters of Grx2-wt, Grx5-wt, Grx5-AS, Grx5-loop, and Grx5-loop/AS under ambient conditions. The curves represent the mean of three independent experiments. Initial cluster concentrations ranged from 50 to 150 µmol l^−1^. Rate constants were obtained from the slope of the curves following linear regression. [FeS]_t_ is the cluster concentration at time point t, [FeS]_*t* = 0_ the initial cluster concentration. Blue curves represents the proteins with the CxxC/S-type loop, brown curves the proteins with the CGFS-type loop, the gray curve Grx5 with the CSYC active site of Grx2. Source data are provided as a Source Data file.

The UV/VIS spectra (Fig. 4a) demonstrate the ability of all proteins to form FeS cluster-bridged holo-complexes. The spectra show the typical absorption bands around 320 and 420 nm. Especially the latter band differs between the Grx classes. We determined the second major peak of holo-Grx2 at 428 nm (using the 1^st^ and 2^nd^ derivatives of the spectra), the peak of holo-Grx5 at 413 nm (dotted vertical lines in Fig. 4a). For the Grx2-loop mutant, this absorption band shifted down to 421 nm, for the Grx5-loop mutant, the band shifted up to 420 nm. Hence, the exchange of the loop structures changed the spectral properties of the holo-protein complexes towards that of the other class. The exchange of the active site in Grx5-AS mutant did not induce notable changes in the absorbance of the FeS cofactor compared to Grx5-wt.

We have also analyzed the stability of the Fe_2_S_2_ clusters under ambient conditions at 25 °C. Clusters decay fitted well with first order kinetics (see the integrated first order kinetics in Fig. 4b). The clusters in Grx2-wt (first order rate constant: (1.1 ± 0.2) × 10^−5^ s^−1^) were more stable than the ones bound to Grx5-wt (*k* = (4.1 ± 0.6) × 10^−5^ s^−1^). This is in accordance with Grx5’s function as FeS transferase. The Grx5-AS mutation stabilized the FeS clusters already (*k* = (1.6 ± 0.3) × 10^−5^ s^−1^); the loop and loop/As mutations in Grx5 stabilized the clusters to essentially the same rate constant as observed for Grx2: Grx5-loop *k* = (9.4 ± 1.7) × 10^−6^ s^−1^, Grx5-loop/AS *k* = (1.2 ± 0.2) × 10^−5^ s^−1^.

### Function of the engineered mutants in FeS biogenesis in vivo

**Fig. 5:**
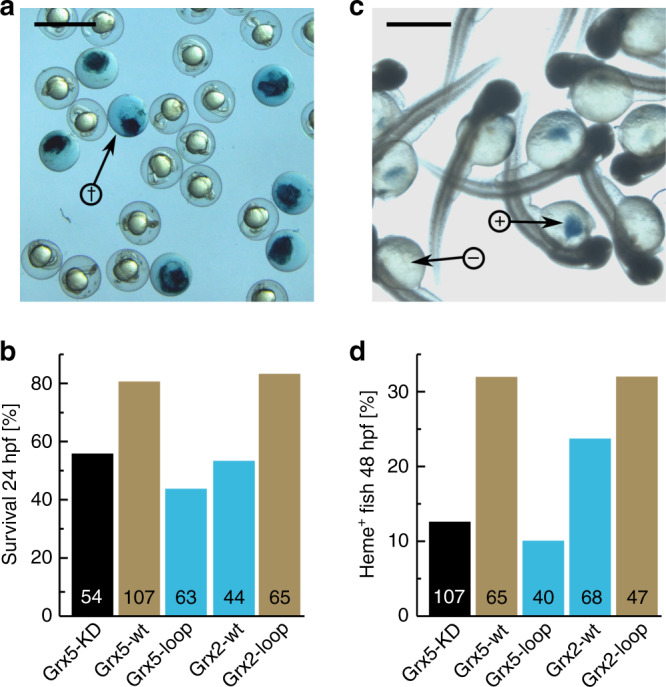
Complementation of the loss of Grx5 in zebrafish by the engineered Grxs. **a** Example image and quantification **b** of dead (stained by methylen blue) and alive zebrafish embryos 24 h post fertilization (hpf). The arrow with cross in **a** points to a dead embryo. Heme-positive staining (**c**) and quantification (**d**) via diaminofluorene in zebrafish embryos 48 hpf. The arrows in **c** point to heme-positive (+) and negative (−) embryos. Zebrafish Grx5 was knocked-down by a specific morpholino (MO) and rescued with mRNA encoding human Grx5-wt, Grx5-loop, Grx2-wt, and Grx2-loop. All mRNAs contained the same standard mitochondrial target sequence. Percentage of survived embryos 24 hpf (**c**) and of heme positive embryos 48 hpf (**d**) was calculated. The bars for CGFS-type loop Grxs are depicted in brown, CxxC/S-type loop Grxs in blue. The numbers (*n*) of the analyzed (independently transfected) embryos were indicated within the bars. The scale bar in **a** measures 2 mm in length, the scale bar in **c** 0.5 mm. The black bar represents the controls, blue bars represents the proteins with the CxxC/S-type loop, brown bars proteins with the CGFS-type loop. Source data are provided as a Source data file.

The Grx5-loop mutant gained oxidoreductase activity, the Grx2-loop mutant lost most of its oxidoreductase activity. What about the in vivo function of the CGFS-type Grxs, i.e. mitochondrial FeS biogenesis? To analyze the functionality of our engineered mutants in vivo, we turned to the zebrafish model. The expression of the endogenous zebrafish mitochondrial CGFS-type Grx5 was silenced using the morpholino technique (see ref. ^14^). The loss of Grx5 decreased the survival of the fish embryos 24 h post fertilization (hpf) from 98% (1 in 52) in our untreated control fish embryos to 56% in the knock-down fish (Fig. 5a, b). Complementation with mRNA for the expression of human Grx5-wt, that is not targeted by the morpholino, rescued the survival rate to 82%. The expression of human Grx2-wt (52% survival) as well as the Grx5-loop mutant (43% survival) with gained oxidoreductase activity could not complement the functional loss of Grx5. The human Grx2-loop mutant, that lost most of its oxidoreductase activity, however, rescued the viability of the Grx5-silenced embryos to the same level as the human Grx5-wt protein (83% survival).

The decreased survival of the Grx5-depleted embryos might be caused by a loss of FeS transfer to the various target proteins. One of these FeS proteins is ferrochelatase, the enzyme that catalyzes the final step in heme maturation^31^. As a result, only 13% of Grx5 knock-down fish could be stained positively for heme at 48 hpf (Fig. 5c, d), whereas 96% (69 of 72 embryos) of control fish were heme positive. Also this phenotype was partially rescued by mRNA encoding either human Grx5-wt or the Grx2-loop mutant (both resulted in 32% heme positive embryos), but not by the human Grx5-loop mutant (10%), and only to a lower extend by human Grx2-wt (24%). Together, these results confirm the gain of function in FeS cluster biosynthesis of the engineered loop mutant of Grx2.

### Alternative conformations of the dimeric holo-complexes

**Fig. 6:**
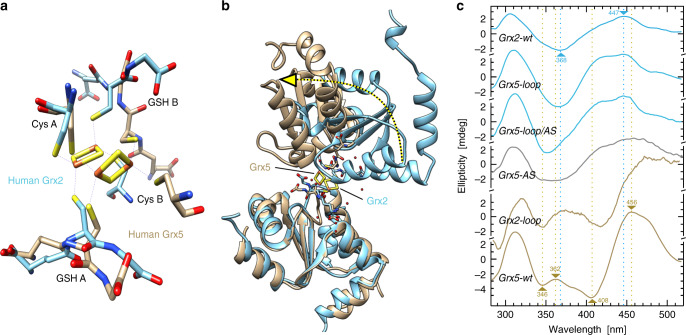
The distinct loop structures determine the conformations of the holo-complexes. **a**, **b** Comparison of the dimeric holo-complexes of the CxxC/S-type Homo sapiens Grx2 (blue, PDB 2HT9) and the CGFS-type Homo sapiens Grx5 (brown, PDB 2WUL). The alternate positions of the Fe_2_S_2_ clusters are indicated (**a**) as well as the rotation of the second monomer induced by the shift and rotation of the FeS cluster (**b**, dotted arrow). The second dimer present in the 2WUL structure was omitted for clarity. **c** Circular dichroism spectra of the wild-type and engineered proteins as indicated. The concentration of the FeS holo-proteins was adjusted to 175 µM using the molar absorptivity of the FeS clusters for all CD spectra recorded. Blue curves represents the proteins with the CxxC/S-type loop, brown curves the proteins with the CGFS-type loop, the gray curve Grx5 with the CSYC active site of Grx2. Source data are provided as a Source Data file.

The structures of the dimeric holo-complexes of Grx2 and Grx5 display profound differences (Fig. 6a, b). The different orientation of the GSH thiol (see also Fig. 2) causes different positions of the FeS clusters bound to the two classes of Grxs (Fig. 6a). This, in consequence, changes the relative orientation of the two protein subunits towards each other. Compared to the Grx2 holo-complex, the second Grx monomer in the Grx5 complex is rotated by ~90° (dotted arrow in Fig. 6b). We hypothesized that the CxxC/S- and CGFS-type loops predefine these different conformations.

Such differences can be assessed using CD spectroscopy. The ellipticity of the holo-complexes in the visible light, where the FeS cofactors absorb light (see Fig. 4a), is a result of the non-chiral cofactor complexed in between the chiral protein ligands. Hence, it also reflects the different orientations of the subunits towards each other. In fact, the spectra of wild-type Grx2 and Grx5 show some profound differences (Fig. 6c). Grx2-wt shows maxima at 305 and 447 nm, and a minimum at 368 nm. Grx5-wt displays maxima at 312, 362, and 456 nm, as well as minima at 346 and 408 nm. Our functional analysis of the proteins suggested that the Grx2-loop and the Grx5-loop mutants should form holo-complexes that reflect the conformations of the other Grx class. As depicted in Fig. 6c, the Grx5-loop mutant shows essentially the same features as Grx2-wt. In particular, it lost the Grx5-specific maximum at 362 nm and minimum at 408 nm. The Grx2-loop mutant displays features that better reflect the characteristics of the Grx5-wt protein than those of its Grx2-wt parent protein, it gained the Grx5-specific maximum in the region of 362 (shifted to 370) nm the minimum in the region of 408 nm (shifted to 420, Fig. 6c). The exchange of the active site in Grx5 resulted in CD properties that lay in between those of the two wild-type proteins.

## Discussion

In general, CxxC/S-type Grxs function primarily in redox regulation and electron supply to metabolic enzymes, CGFS-type Grxs in FeS cluster biogenesis and iron trafficking^32,33^. The human CxxC/S-type Grx2 and the CGFS-type Grx5 can both form the dimeric or tetrameric holo-complex with the bridging Fe_2_S_2_ cluster that is ligated by the two N-terminal active site thiols and the thiols of two non-covalently bound GSH molecules^7,9,22^; both proteins are localized primarily in the matrix of mitochondria.

For Grx2, the Fe_2_S_2_ cluster was discussed to serve as redox sensor of the protein for its activation in response to various redox signals^5^. Disassembly of the holo-complex generates the apo-form of Grx2 that is enzymatically active in both the monothiol and dithiol reaction mechanisms and can be reduced by both GSH and thioredoxin reductase^12,34^. The protein protects from redox insults, e.g. induced by the anti-cancer drug doxorubicin^35–37^, likely by catalyzing the reversible glutathionylation of membrane protein complexes inside mitochondria to regulate their function^38,39^. Moreover, the disassembly of the Grx2 Fe_2_S_2_ cluster by nitric oxide leads to the formation of dinitrosyl-diglutathionyl-iron complexes, and thus to the detoxification of nitric oxide and the protection of oligodendrocytes against inflammation-induced cell damage^40^. Grx5, on the other hand, is basically inactive as oxidoreductase. Instead, the protein has essential functions in the biogenesis of FeS proteins, a function that seems to be conserved in all eukaryotic cells^15–17,19,41–43^. At present, the most widely accepted function of the CGFS-type Grxs is to serve as transferases of their Fe_2_S_2_ clusters from the assembly machinery to target proteins^17,33^. Recent structural studies suggest that holo-Grx5 works as a metallo-chaperone preventing the Fe_2_S_2_ cluster to be released in solution and to form a transient, protein-protein intermediate with target proteins receiving the Fe_2_S_2_ cluster^44^. Based on these divergent functions, the consolidation of the CGFS-type and CxxC/S-type Grxs into one functional class as well as the classification of the CGFS-type Grxs as oxidoreductases may have to be revised.

The Grx5 mutant with the CxxC/S-type loop preceding the active site analyzed here (Grx5-loop) showed a clear increase in activity as reductant of the roGFP2 protein as well as in the HED assay (Table 1, Fig. 3). An even more pronounced increase in activity was demonstrated in the catalysis of the oxidation of the roGFP2 model substrate. In fact, here it exceeded the activity of the reference protein. Why is the oxidation reaction preferred? Both the reduction of roGFP2 as well as the HED assay require the reaction of the Grx-S-SG intermediate with a second molecule of GSH (reaction 1 reverse and reaction 6, see above), this second GSH binding site has only been established recently^10^. The additional exchange of the CGFS to the CSYC active site further increased the activity of the loop mutant. This was also true for the de-glutathionylation of the other proteins analyzed, the Grx5 double mutant was always the most efficient. Our results suggest that the Gly-Phe residues prefer a conformation that hinders the interaction with the second GSH molecule, likely by blocking or shifting the access to the mixed Grx-S-SG disulfide that has to be attacked in a 180° angle to form the tertiary intermediate required for the thiol-disulfide exchange reaction^28^. Likely, more features of the CGFS-type Grxs disfavor this second interaction, or— as suggested before—more features are required to fully facilitate the activation of the second GSH molecule for the attack of the disulfide^10^.

The importance of the strictly conserved lysyl residue for the function of both classes of Grxs has been addressed before. Yeast CxxC/S-type Grx8p, which has an alanyl residue at the strictly conserved lysyl position, exhibited very low activity in standard assays^45,46^. Furthermore, mutations of the conserved lysyl residue in the CGFS-type human Grx5 or *A. thaliana* GrxS15 decreased the activity of various FeS-proteins indicating their decreased functionality as FeS cluster transfer proteins^19,47^.

Our study provides evidence that the CGFS-type Grxs evolved in a way that hampers if not prevents the formation of the Grx-S-SG mixed disulfide intermediate between the N-terminal active site thiol and glutathione and thus impairs activity as oxidoreductases. Considering their primary function as Fe_2_S_2_ cluster carrier proteins, this would be a logical evolution as the thiol-disulfide oxidoreductase activity competes with the formation of the FeS holo-complex. The decreased stability of the Fe_2_S_2_ cluster coordinated by Grx5 (Fig. 4b) is the second consequence of the direct interaction of the amino group of the lysyl residue in the CGFS-type Grx loop with the GSH thiol, as demonstrated by the Grx5-loop mutant (Fig. 4b), that forms a considerable more stable holo-complex. The lower stability of the holo-complex may be seen as a prerequisite for a thermodynamically more favorable cluster transfer, as suggested, for instance, for *E. coli* CGFS-type Grx4 before^23^. The third consequence of the CGFS-type loop is a shift and rotation in the location of the Fe_2_S_2_ cluster in the holo-complex due to the rotation of the GSH cysteinyl side chain (see Figs. 1d, 2a, b, and 6a, b). This shift profoundly affects the orientation of the two monomers in the holo-complex towards each other (Fig. 6a). As discussed elsewhere, this may be a prerequisite for an efficient transfer of the Fe_2_S_2_ cluster^44^. Our CD spectroscopy results confirm the re-orientation of the two monomers in the engineered mutants towards the wild-type of the converse proteins. Our in vivo results in the zebrafish (Fig. 5) confirm the gain of FeS cluster transfer function in the Grx2-loop mutant, even though our rescue efficiency was relatively low compared to the one published before. Wingert et al.^14^ had already pointed out the decrease in rescue efficiency with Grxs from different species. In addition, we changed the mitochondrial transit sequence of all proteins to one from *Neurospora crassa*, to exclude effects caused by different mitochondrial translocation efficiencies. It is likely that this further decreased rescue efficiency. The differences demonstrated using nearly 800 individual fish, however, support our conclusion that the loop is the major determinant discriminating between oxidoreductase activity and FeS transferase activity.

We suggest a refined model of Grx actions (summarized in Supplementary Fig. 9). As described earlier, all Grxs exhibit a similar binding site for the first GSH residue that is composed of four conserved motifs, see^10,24^. This binding site allows the formation of the mixed disulfide Grx-S-SG intermediate that is a prerequisite for oxidoreductase activity. It does, however, also allow the non-covalent binding of GSH, a prerequisite for FeS cluster ligation. The major determinant of cluster ligation is the absence of a prolyl residue in the second position of the CxxC/S active site^7^. The extended loop preceding the active site, as well as the presence of a glycyl residue in position 2 of the active site in CGFS-type Grxs distinct two modes of GSH binding to this first binding site. One with the GSH thiol pointing towards the N-terminal active site Cys, in redox active Grxs, the second with the thiol orientation shifted away from the active site, in FeS transferring Grxs. The second GSH interaction site is required for the reduction of the Grx-S-SG intermediate by a second GSH molecule. The attack on the mixed disulfide requires access to the bond in a 180° angle to form the required tertiary intermediate^28^. The CGFS active site appears to partially block this access, however, our data also suggest that more features are required to fully facilitate this reaction.

In a recent study, Liedgens et al.^48^ also addressed the structural differences between the two main classes of glutaredoxins with focus on the mechanistic understanding of the oxidoreductase functions of Grxs. Using *S. cerevisiae* Grx7, a CxxS-Type Grx, as model protein, the authors identified several key residues and quantified their contribution to the interactions with both the first and second molecule of GSH^48^ (also see Supplementary Fig. 9). Together, these studies provide a mechanistic explanation for both the lack of redox activity of the CGFS-type Grxs as well as their higher propensity for Fe_2_S_2_ cluster transfer by affecting both the stability of the holo-complex as well as their interaction with target proteins. A small shift of only 0.2 nm in the position of the ε-amino group of a strictly conserved lysyl residue at the beginning of distinct loops separates the two Grx classes. These different conformations affect the position and reactivity of the GSH cysteinyl thiol, the orientation of the ligated Fe_2_S_2_ cluster, and the orientation of the monomers in the dimeric holo-complex. While the primary effect may seem small, the ripple effects profoundly control the functions of the proteins.

## Methods

### Materials

Chemicals and enzymes were purchased at analytical grade or better from Sigma-Aldrich (St. Louis, MO, USA). GSNO was synthesized as described by Hart et al.^49^.

### Structural analysis

Structures were acquired from the RSCB PDB Protein Data Bank [http://www.rcsb.org], ligands and water molecules were removed using Pymol. The most representative structure of NMR ensembles was identified using UCSF Chimera^50^. All pre-oriented protein structures were used to further compute the electrostatic potential and the iso-surfaces of the electrostatic potential. PQR files were generated using pdb2pqr^51^ applying the amber force-field. VMD (visual molecular dynamics)^52^ and APBS (Adaptive Poisson-Boltzmann Solver)^53^ were used to compute the electrostatic potential in an aqueous solution containing 150 mM mobile ions at a temperature of 298 K.

### Molecular dynamics simulations

For Grx2, PDB 2FLS was used for both simulations of the apo-protein and GSH complex. For Grx5, 2MMZ was used for the apo protein and 2WUL for the GHS-bound complex. All structures were prepared for MD simulations using UCSF Chimera^50^ including the removal of all water molecules, non-complexed ions, and crystallization additives; adding any missing loops and residues via MODELLER^54^ and using Dunbrack rotamer library^55^. Side chains with alternate location were fixed by selecting the highest-occupancy conformers. Preparation of the GSH complexes included parametrization of γ-glutamate residue using ACPYPE^56^, with GAFF force field^57^ and AM1-BCC^58^ partial atomic charges assigned. All simulations were performed using Gromacs 2016.3^59^. The protein was parameterized using the AMBER99SB-ILDN force field, with the TIP3P water model^60^. Box distance was set to 1 nm and periodic boundary conditions were applied. The box was solvated and Na^+^ and Cl^−^ ions were added at 0.1 M concentration to maintain unit neutrality. The solvated systems were energy minimized and equilibrated. The minimization ran using steepest descent for 1000 cycles followed by the conjugate gradient. Energy step size was set to 0.001 nm and the maximum number of steps was set to 50,000. The minimization was stopped when the maximum force fell below 1000 kJ/mol/nm using the Verlet cut-off scheme. Treatment of long-range electrostatic interactions was set to Particle Mesh-Ewald (PME)^61^, and the short-range electrostatic and van der Waals cut-off set to 1.0 nm. Following the energy minimization, heating to 300 K was performed for 20 ps with a time step of 2 fs and position restraints applied to the backbone in a NVT ensemble. The constraint algorithm used was LINCS, which was applied to all bonds and angles in the protein^62^. The cut-off for non-bonded short-range interaction was set to 1.0 nm with the Verlet cut-off scheme. The temperature coupling was set between the protein and the non-protein entities by using a Berendsen thermostat, with a time constant of 0.1 ps and the temperature set to reach 300 K with the pressure coupling off. Pressure equilibration was run at 300 K with a Parrinello-Rahman barostat on and set to 1 bar^63^ in a NPT ensemble. The equilibration trajectories were set to 5 ns (discarded from the analysis), and the production MD simulations were performed for 100 ns. Each simulation was run in triplicates. Analysis of the trajectories was performed using GROMACS tools, including root-mean-square deviation (RMSD) to assess overall stability, per-residue root-mean-square fluctuation (RMSF) to assess the local flexibility, and all the distributions of interatomic distances and angles.

### Cloning of expression constructs

The Homo sapiens Grx2 (Grx2c) expression construct was described in^64,65^, the Arabidopsis thaliana GrxC1 construct in^10^. Homo sapiens Grx5 was amplified from human cDNA by PCR using DyNAzyme EXT DNA polymerase (Thermo Scientific, Weltham MA, USA) and ligated into the pGEMT plasmid (Promega, Madison WI, USA). Primers were designed to insert restriction sites for NdeI (forward 5′-CACACACATATGGGCTCGGGCGCGGGC-3′) and BglII (reverse 5′-CACACAAGATCTTCACTTGGAGTCTTGGTCTTTCTTTTCATCTAAAAGG-3′). The insert was ligated into the expression vector pET15b (Merck, Darmstadt, Germany). Mutations of the amino acid sequence and amplification of the plasmids were performed by rolling circle PCR. We generated the mutants using the indicated primers and the reversible complementary counterparts if not stated otherwise. Grx5-loop: (5′-GGACAAGGTGGTGGTCTTCCTCAAGCCCCAGTGCGGCTTCAGCAACG-3′). For Grx2-loop: 5′-GGGACGCCGGAGCAGACATCCTGTTCTTACTGTACAATGG-3′ as forward and 5′-CTGCTCCGGCGTCCCTTTTGAGAAAATCACCACACAATTATCAGAAATTG-3′ as reverse primer. The CGFS active site in Grx5 was replaced for CSYC using 5′-CAGCCCCAGTGCAGCTACTGCAACGCCGTG-3′ for the wild-type (Grx5-AS) and 5′-CAAGCCCCAGTGCAGCTACTGCAACGCCGTG-3′ for the Grx5-loop mutant (Grx5-loop/AS). All constructs and mutations were verified by sequencing (Microsynth Seqlab, Göttingen, Germany).

### Recombinant expression and purification

Homo sapiens Grx2, Grx5, their mutants, E. coli IscS, roGFP2, D. rerio Sirt1, and AtGrxC1 were expressed as His-tagged proteins and purified via immobilized metal affinity chromatrography^7,10,66^. SDS-PAGE was performed using pre-casted TGX stain-free gels (4–20%, BioRad, Hercules CA, USA) and imaged according to the manufacturers’ instructions. Protein concentration was determined at 280 nm (*ε*_Grx5_ = 11,585 and *ε*_Grx2_ = 7,450 M^−1^ cm^−1^).

### Differential scanning fluorimetry (thermofluor assay)

The thermal stability of wild-type and mutant proteins were assayed using the thermofluor assay^67^. In brief, 10 µM protein, re-buffered in PBS were mixed with SYPRO Orange (1:100 diluted, Sigma-Aldrich) and heated in a CFX96 Real Time System from BioRad in 0.5 K increments from 20 to 95 °C in one hour. The increase in fluorescence due to binding of the dye to hydrophobic regions exposed during denaturation was recorded using the instrument’s “FRET”-settings. The curves represent the average of seven replicates.

### Kinetics of the reduction and oxidation of roGFP2 by Grxs

Interaction of Grxs with roGFP2 was analyzed in vitro by ratiometric time-course measurements on a fluorescence plate reader^68^ (Clariostar; BMG Labtech, Offenburg, Germany) with excitation at 390 ± 10 and 480 ± 10 nm and detection of emitted light at 520 nm with a bandwidth of 10 nm. In all, 0.1 M potassium phosphate buffer pH 7.8 containing 1 μM roGFP2 and the respective Grx. Ratiometric time-course measurements were carried out with initially oxidized or reduced roGFP2, respectively. For the latter, the protein was reduced with 10 mM DTT for at least 20 min. The remaining DTT was removed by desalting spin columns (Zeba Spin Desalting Columns, Thermo Scientific). For interaction analysis with oxidized roGFP2, GSH (in 0.1 M potassium phosphate buffer, pH 7.0) was included to a final concentration of 2 mM. When working with reduced roGFP2, a highly negative redox potential of the glutathione buffer was maintained by addition of 10 U glutathione reductase and 100 μM NADPH. For oxidation of roGFP2, 40 μM GSSG were included in the wells. H_2_O_2_ and DTT were used at a final concentration of 10 mM to preset roGFP2 to the fully oxidized and fully reduced state, respectively, and determine maximum and minimum fluorescence ratios of roGFP2 as reference values. A basal background fluorescence of buffer or buffer containing 100 μM NADPH was subtracted from fluorescence reads for all samples. The *k*_cat_ calculated was defined as the specific initial rate of roGFP2 oxidation and reduction, respectively, at 1µmol·l^−1^ substrate concentration.

### HED assay

The hydroxyethyl disulfide (HED) assay^34^ was performed in a 96-well plate format. The final reaction mixtures of 200 µl contained 100 mM potassium phosphate buffer (pH 7.8), 200 µM NADPH, 1 mM GSH, 3 × 10^−3^ g l^−1^ glutathione reductase from yeast (G3664, Merck, Darmstadt, Germany), and variable concentrations of HED (0–1 mM). The concentration of the Grxs was optimized for all proteins individually and lay between 0.5 and 62.5 µg ml^−1^. The assay was run for 20 min. The linear range of the decrease in absorption was determined for each reaction individually.

### Analysis of protein de-glutathionylation

De-glutathionylation using single substrates follows a protocol published in ref. ^66^. 30 µM of BSA (Sigma) or recombinant D. rerio Sirt1 were reduced with 10 mM DTT, desalted (Zeba spin, Thermo Fisher), glutathionylated with 150 µM Di-Eosin-GSSG (kind gift of Arne Holmgren), and desalted again. 10 µM substrate were incubated for 15 min at 37 °C with 1 mM GSH and ±50 µM pre-reduced Grx mutants. After SDS-PAGE, fluorescent GSH was visualized by UV-light and proteins were stained with Coomassie. For the de-glutathionylation of whole lysates, 40 µg HeLa cell extract was incubated with 5 mM GSSG. After desalting (PD10, GE Healthcare), cell extracts were incubated with 1 mM GSH and ±60 µM Grx mutants for five minutes. Proteins were separated by SDS-PAGE, glutathionylated proteins were visualized with anti-GSH antibodies (#ab19534, abcam, dilution 1:1000) after Western blotting.

### Determination of free thiols

Proteins were reduced (50 mM DTT, 30 min), re-buffered in TE buffer (100 mM Tris/HCl and 2 mM EDTA) using NAP-5 columns (GE Healthcare) and incubated with 10 mM GSSG before alkylation with 10 mM NEM for 20 min each. Next, free thiols were assayed (see below) yielding the values for the oxidized and alkylated samples. Part of the samples were re-reduced (50 mM DTT, 30 min) and re-buffered again in TE to remove the excess of DTT. Next, the samples were incubated with 1 mM DNTB and 1% SDS in TE buffer for 15 min. Absorbance was measured at a wavelength of 412 nm using a 96-well plate reader (Tecan Infinite M200). The amount of free thiols was calculated using the molar absorption coefficient of *ε* = 14,150 M^−1^ cm^−1^ and normalized to the amount of protein in the sample.

### Reconstitution and stability of FeS-clusters

Approximately 100 µM of the recombinant redoxins were incubated at RT under argon atmosphere in sodium phosphate buffer pH 8.0, including 200 mM NaCl, 5 mM DTT, 1 mM GSH, 200 µM Fe(NH_4_)_2_ (SO_4_)_2_ 250 µM cysteine, 10 µM pyridoxal phosphate, 1 µM *E. coli* IscS. After 2 h, the proteins were desalted using Zeba Spin columns and UV/Vis-spectra were recorded. Kinetics of FeS-cluster disassembly were followed at 420 nm at 25 °C. UV/Vis-spectra and kinetics were recorded by an UV-1800 spectrometer (Shimadzu Kyoto, Japan). FeS clusters were quantified as outlined below. All experiments were repeated independently at least three times. Initial cluster concentrations ranged from 80 to 250 µmol l^−1^. Rate constants were obtained from the slope of the integrated first order kinetics: ln([FeS]_t_·[FeS]_*t* = 0_^−1^)  = *k*·t, where [FeS]_t_ is the cluster concentration at time point t, [FeS]_*t* = 0_ the initial cluster concentration, *k* the first order rate constant and t the time in seconds.

### Determination of iron content

The FeS content per Grx dimer was calculated from UV-vis spectra using the molar absorptivity of the cluster in 100% dimeric Grx2 at 430 nm as determined in ref. ^69^ (*ε*_430_ = 3260 M^−1^ cm^−1^). Protein bound iron was quantified according to ref. ^70^. In brief, 200 µM of in vitro freshly reconstituted proteins were mixed with 0.5-fold volumes of 0.6 M HCI and 0.142 M KMnO_4_ and incubated for 2 h at 60 °C. Next, the solution was incubated for 30 min at room temperature with 0.1-fold of the original sample volume of 6.5 mM ferrozine, 13.1 mM neocuproine 6, 2 M ascorbic acid, and 5 M ammonium acetate. The iron-ferrozine complex was quantified photometrically at 562 nm.

### Zebrafish

Zebrafish (AB/TL wild-type line, ZFIN ID: ZDB-GENO-960809-7; ZDB-GENO-990623-2) were kept in standard conditions (14 h light and 10 h dark, 28 °C, pH 7.2, 500 µS). Eggs were, in accordance with national law, obtained by natural spawning. The morpholino targeting Grx5 (5-′CTGTCGACCTAAAAACGCTATTCAT-3′, ZFIN ID: ZDB-MRPHLNO-051203-1)^14^ was synthesized by Gene Tools and diluted in water to a concentration of 3 mM. Human wild-type and mutant Grx2 and five cDNAs were modified with the mitochondrial transit sequence of *Neurospora crassa* subunit 9 of the F1F0 ATPase (aa 1–69) and then cloned into the pGEM-T-Easy Vector (Promega) 3′ of the T7 promotor. Capped mRNA was generated with the mMessage/mMachine Kit (Ambion) and subsequently polyadenylated with the Poly(A) Tailing Kit (Invitrogen). Survival rate was determined after injection of 1.77 nl of a 1/8 dilution of the Grx5 morpholino and of 60 pg capped polyadenylated mRNA into embryos at the one cell stage. Dead embryos were visualized by accumulation of methylen blue present in the tank water at a concentration of 2 mg/l and counted 24 hpf. Heme staining was performed after injection of 1.77 nl of a 1/10 dilution of the morpholino together with 120 pg mRNA 48 hpf. Non-injected embryos served as controls. For dechorionation, embryos were incubated for 15 min with 0.5 mg/ml Pronase (Sigma). Staining with 2,7-Diaminofluorene (Sigma) was performed as described previously^21^. All zebrafish experiments were performed in accordance with the German and European animal welfare legislation. According to the EU Directive 2010/63/EU on the protection of animals used for scientific purposes, early life-stages of zebrafish are not protected as animals until the stage of being capable of independent feeding, i.e. 5 days post fertilization.

### CD spectroscopy

CD spectra were recorded in 300 mM NaCl, 50 mM sodium phosphate, 1 M sucrose, pH 8 with the FeS holo-proteins at 175 µM concentration in a Jasco J-810 spectropolarimeter. The concentrations of the holo proteins were determined based on the molar absorptivity of the FeS-holo proteins at 430 nm (*ε*_430_ = 3260 M^−1^ cm^−1^)^69^. Buffer-only spectra were subtracted. A standard sensitivity of 100 mdeg was used with a data pitch of 1 nm, a scanning speed of 50 nm/min, and a band width of 1 nm. Three spectra were accumulated for each sample.

### Software

All numerical calculations (spectra, kinetics) were performed and visualized using grace [https://plasma-gate.weizmann.ac.il/Grace/]. Blot pictures were normalized using ImageLab 5.1 (Biorad) and Gimp [https://www.gimp.org/]. Densiometric analyses were performed using ImageLab and ImageJ [https://imagej.net]. Structures were depicted using UCSF Chimera^49^. Picture panels and reaction schemes were generated using Inkscape [https://inkscape.org/].

### Reporting summary

Further information on research design is available in the Nature Research Reporting Summary linked to this article.

## Supplementary information

Supplementary Information

Peer Review

Reporting Summary

## Data Availability

The data that support this study are available by the corresponding author upon reasonable request. Most of the data that support the findings of this study are available within the paper and its supplementary material. Source data are provided with this paper.

## Source data

Source Data
