# Supplementary material for: Molecular basis for the distinct functions of redox-active and FeS-transfering glutaredoxins
Source: Nat Commun. 2020 Jul 10;11:3445. doi: 10.1038/s41467-020-17323-0 (PMC7351949; doi:10.1038/s41467-020-17323-0)
Supplement: Supplementary file 1 — Supplementary Information [file 41467_2020_17323_MOESM1_ESM.docx]

# Supplementary material to:

# **Molecular basis for the distinct functions of redox-active and FeS-transfering glutaredoxins**

Daniel Trnka*,1, Anna D. Engelke*,2, Manuela Gellert*,1, Anna Moseler3,4, Md Faruq Hossain1, Tobias T. Lindenberg5, Luca Pedroletti3, Benjamin Odermatt5, João V. de Souza6, Agnieszka K. Bronowska6, Tobias P. Dick^7^, Uli Mühlenhoff8, Andreas J. Meyer3,, Carsten Berndt2, and Christopher Horst Lillig1

Affiliations: (1) Institute for Medical Biochemistry and Molecular Biology, University Medicine, University of Greifswald, Greifswald, Germany, (2) Department of Neurology, Medical Faculty, Heinrich-Heine University Düsseldorf, Germany, (3) Institute of Crop Science and Resource Conservation, University of Bonn, Germany, (5) Institute of Neuroanatomy, University Clinics, University of Bonn, Germany, (6) Chemistry, School of Natural and Environmental Sciences, Newcastle University, NE1 7RU Newcastle, UK, (7) Division of Redox Regulation, DKFZ-ZMBH Alliance, German Cancer Research Center (DKFZ), Heidelberg, Germany, and (8) Institute for Cytobiology and Cytopathology, Philipps University Marburg, Germany.

Corresponding author: Christopher Horst Lillig, University Medicine, Institute for Medical Biochemistry and Molecular Biology J.03 33, Ferdinand-Sauerbruch-Straße, DE-17475 Greifswald, Germany, phone: +49 3834 865407, fax: +49 3834 865402, e-mail: [horst@lillig.de](mailto:horst@lillig.de)

* DT, ADE, and MG contributed equally to this work

¶ present address: UMR 1136 Interactions Arbres/Microorganismes, Université de Lorraine, Vandoeuvre-lès-Nancy, France

# Supplementary table 1

Mutants of *Homo sapiens* Grx2 and Grx5 constructed. The numbering refers to the full length sequences including mitochondrial transit signals.

| protein | mutations | name |
| --- | --- | --- |
| Grx2 | 74-**KTS**→**K**GTPEQ**TS** | Grx2-loop |
| Grx5 | 59-**K**GTPEQ**PQ**→**KPQ** (Δ 60-64) | Grx5-loop |
| Grx5 | 67-**C**GFS→**C**SYC | Grx5-AS |
| Grx5 | 59-**K**GTPEQ**PQ**→**KPQ** (Δ 60-64) + 67-**C**GFS→**C**SYC | Grx5-loop/AS |

# Supplementary table 2

Fe/S and iron content in freshly reconstituted and re-buffered FeS Grxs. All data are shown as mean. FeS content from spectra: n=3 biological replicates, ± sd; FeS content colorimetric: n=2 ± absolute deviation. Source data are provided as a Source Data file.

| Protein | FeS content | Fe content |
| --- | --- | --- |
|  | (from spectra) | (colorimetric) |
|  | Mol per Mol Grx (dimer) | Mol per Mol Grx (monomer) |
| Grx2-wt | 0.69 ± 0.14 | 0.72 ± 0.03 |
| Grx2c-loop | 0.64 ± 0.14 | 0.62 ± 0.00 |
| Grx5-wt | 0.72 ± 0.26 | 0.73 ± 0.01 |
| Grx5-AS | 0.68 ± 0.21 | 0.72 ± 0.10 |
| Grx5-loop | 0.65 ± 0.17 | 0.67 ± 0.03 |
| Grx5- loop/AS | 0.69 ± 0.19 | 0.79 ± 0.04 |

# Supplementary figure 1

**
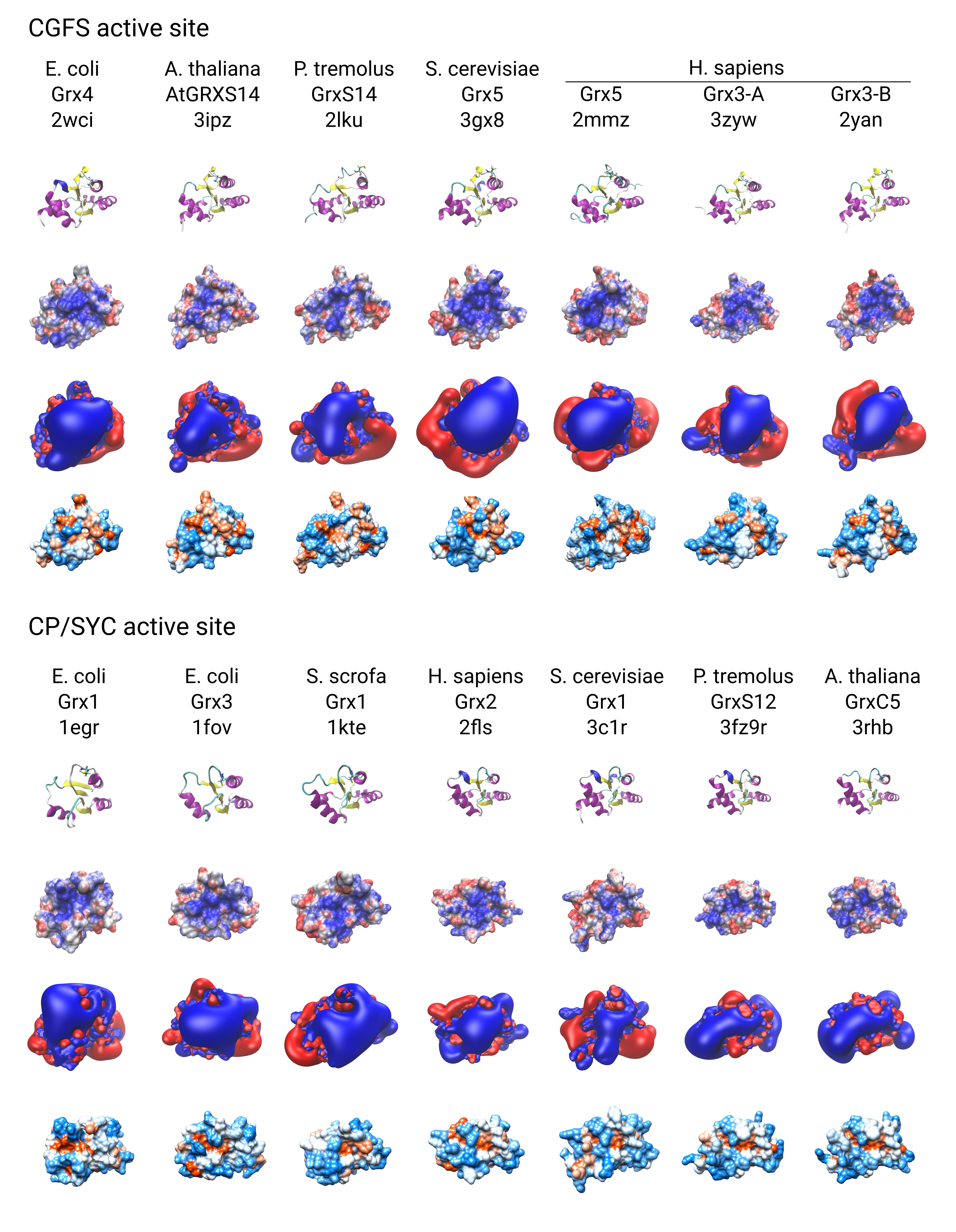
**

**Supplementary Figure 1 – Electrostatic properties of various CGFS-type and CxxC/S-type Grxs.** The electrostatic potential mapped to the surface is pictured with a scale from -100 mV (red) to 100 mV (blue). The isosurfaces of the electrostatic potential are represented in blue (+1 KT/e = 25.8 mV) and red (-1 KT/e = -25.8 mV). Models of the secondary structure, including the N-terminal active site cysteine, the electrostatic potential, the isosurface of the electrostatic potential, and the hydrophobicity (light blue hydrophobic, orange hydrophilic) of the surface are depicted for each protein structure with indicated protein data bank (pdb) entry number.


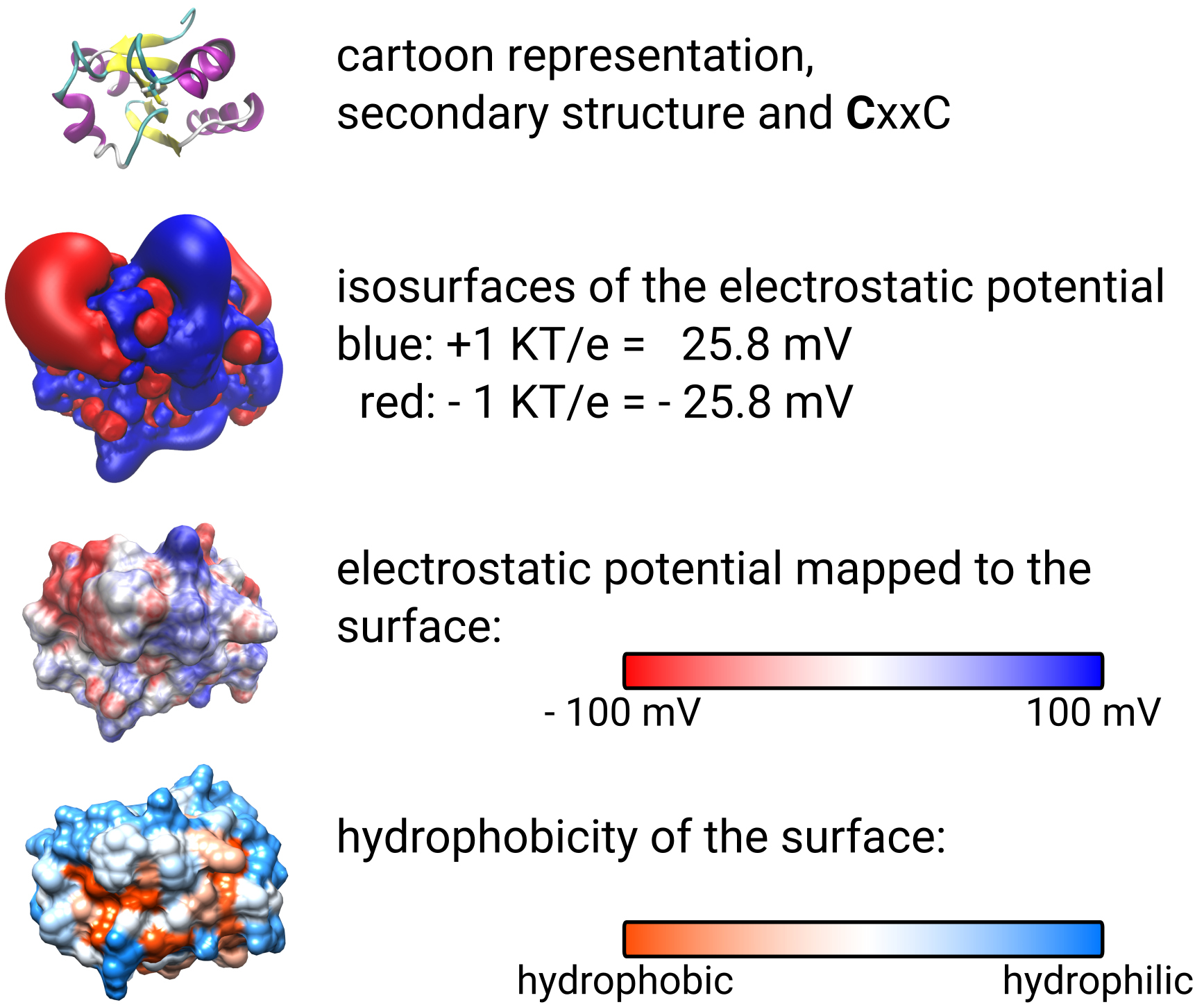


# **Supplementary figure 2**

**
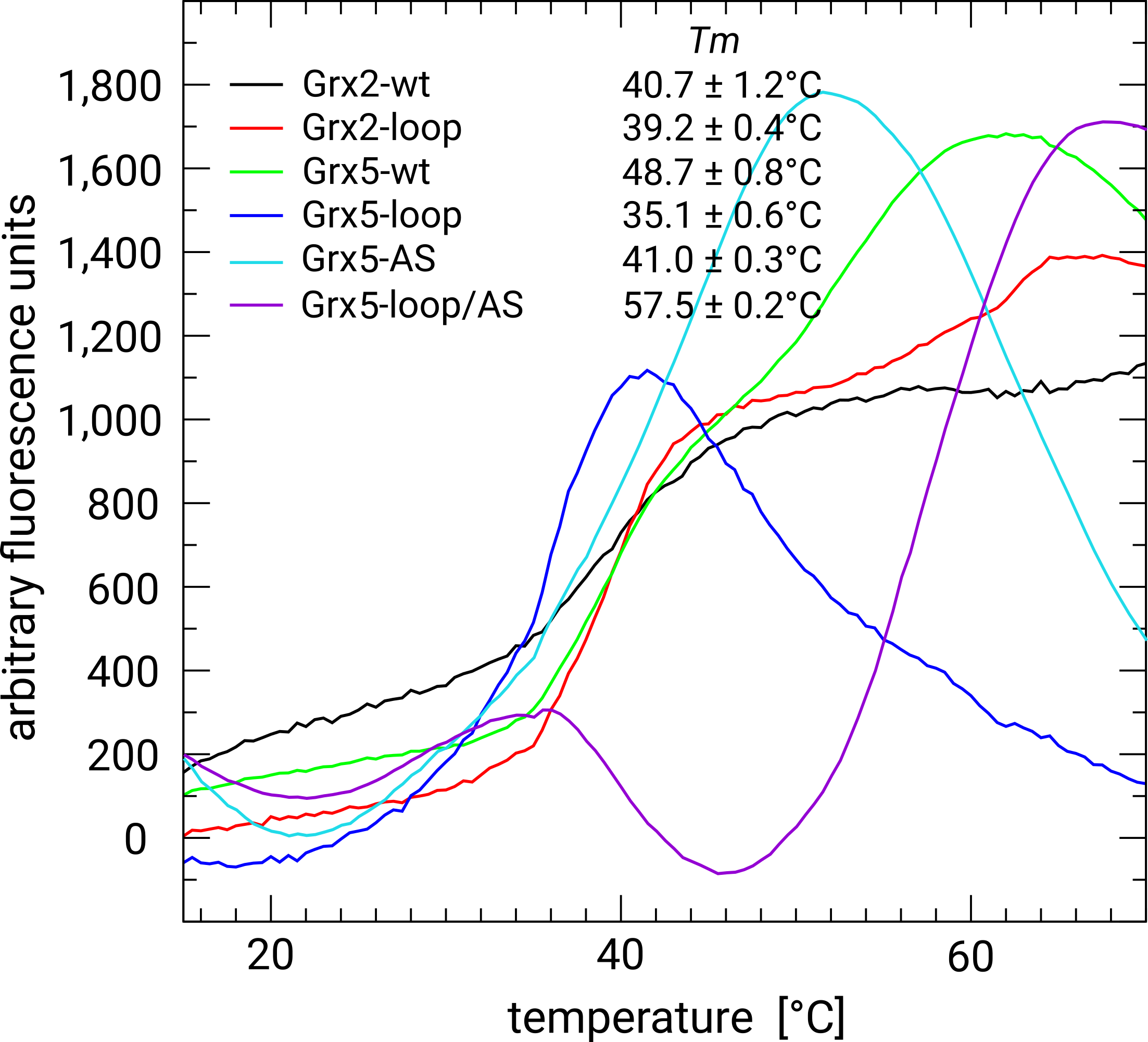
**

**Supplementary Figure 2– Thermal stability of the recombinant wild-type and mutant proteins.** Differential scanning fluorimetry (thermofluor assay) – 10 µM protein were incubated with SyproOrange dye and heated in increments 1K per minute. Binding of the dye to the hydrophobic surfaces exposed during denaturation releases the quenching of the dye’s fluorescence in aqueous solutions. The curves are the average of n=7 biological replicates, the Tm was given as mean ± sd. Colour code: black Grx2-wt, red: Grx2-loop, green: Grx5-wt, dark blue: Grx5-loop, light blue: Grx5-AS, violet: Grx5-loop/AS. Source data are provided as a Source Data file.

# Supplementary figure 3


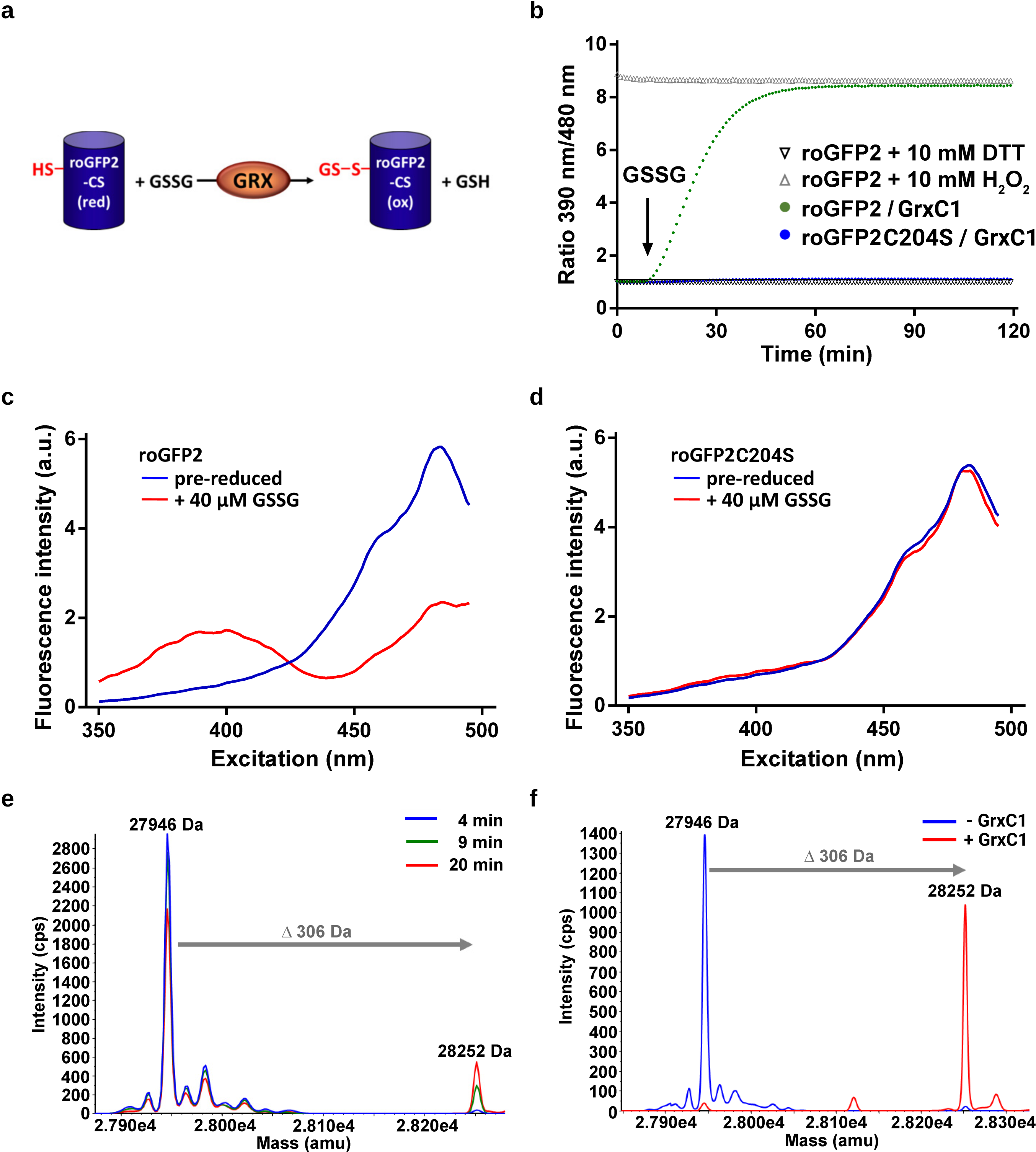


**Supplementary Figure 3 – Mechanism of oxidation of the roGFP2 sensor.** The roGFP sensor is oxidized via a glutathionylated intermediate (b, e-f), however, the ratiometric change of excitation properties only occurs after formation of the intra-molecular disulfide (c-d). Trapping of glutathionylated intermediates of glutaredoxin-mediated roGFP oxidation. (**a**) Glutaredoxin-mediated glutathionylation of single-cysteine roGFP2-C204S(roGFP2CS). (**b**) Time course for the Grx-mediated oxidation of roGFP2 and roGFP2-C204S triggered by addition of 40 µM GSSG (arrow). All data were normalized to the fluorescence ratios of DTT-treated proteins. (**c,d**) Excitation spectra of roGFP2 (**c**) and roGFP2-C204S (**d**) before and after oxidation by 40 µM GSSG in the presence of GrxC1. All proteins were pre-reduced by DTT, which was removed on a desalting column prior to the measurement. Spectra were collected with emission of 520 ± 5 nm (**e,f**) Identification of glutathionylated roGFP-C204S by ESI-Q-TOF analysis. Samples were injected either at different times after incubation of 5 µM roGFP2-C40S with 2 mM GSSG at pH 7.0 in the absence (**e**) or in the presence (**f**) of 5 µM Grx for 20 min. Molecular masses were determined with a precision of ±2 Da. For the colour coding, see figure. Source data are provided as a Source Data file.

# Supplementary figure 4

**
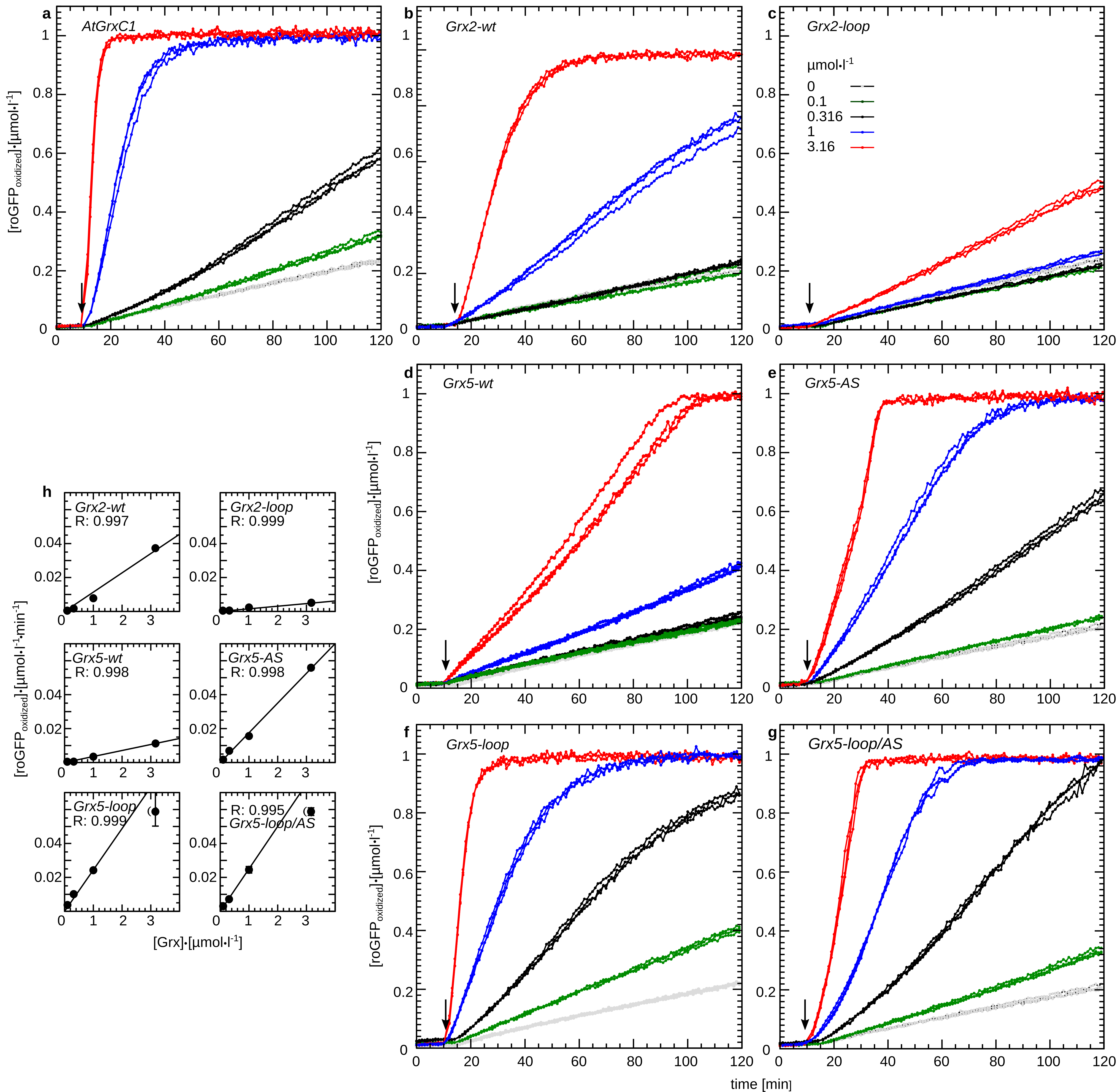
**

**Supplementary Figure 4 – Activity of the Grxs in the oxidation of roGFP.** (**a-g**) The curves show the oxidation of roGFP versus time. All Grxs were tested at 0.1 (green), 0.316 (black), 1 (blue), and 3.16 µmol·l^-1^ (red), respectively; n=3 each. The background reaction without added Grx is depicted in gray. The fluorescence ratios measured for fully oxidized roGFP2 in the presence of 10 mM H_2_O_2_ was set to 100 %, i.e. 1 µmol·l^-1^, the ratios measured for fully reduced roGFP following reduction by DTT was set to 0 % oxidized protein, i.e. 0 µmol·l^-1^. All data were normalized accordingly. GrxC1 from Arabidopsis thaliana (top left) was included as positive control in each plate assay. The arrow marks the time point when the assays were started by addition of GSSG. (**h**) Concentrations of the Grxs used versus activity. All plots fitted well with the linear regression (the correlation coefficient was added in the figures). The points in parenthesis were omitted from the analyses. Data shown as mean ± sd (3 replicates). For the colour coding, see figure. Source data are provided as a Source Data file.

# Supplementary figure 5

**
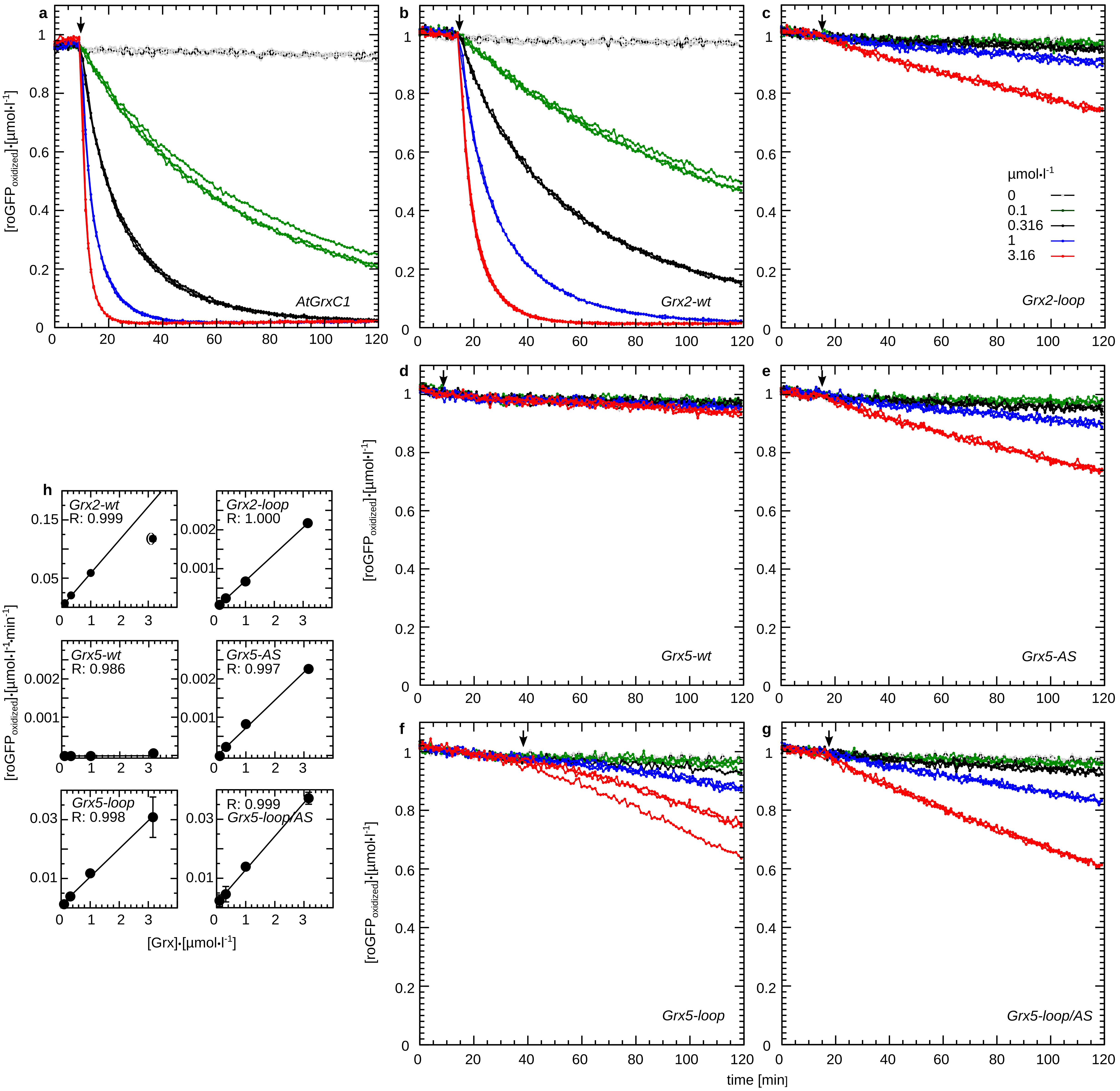
**

**Supplementary Figure 5 – Activity of the Grxs in the reduction of roGFP.** (**a-g**) The curves show the reduction of roGFP versus time. All Grxs were tested at 0.1 (green), 0.316 (black), 1 (blue), and 3.16 µmol·l^-1^ (red), respectively; n=3 each. The background reaction without added Grx is depicted in gray. The fluorescence ratios measured for fully oxidized roGFP2 in the presence of 10 mM H_2_O_2_ was set to 100 %, i.e. 1 µmol·l^-1^, the ratios measured for fully reduced roGFP following reduction by DTT was set to 0 % oxidized protein, i.e. 0 µmol·l^-1^. All data were normalized accordingly. GrxC1 from Arabidopsis thaliana (top left) was included as positive control in each plate assay. The arrow marks the time point when the assays were started by addition of GSH. (**h**) Concentrations of the Grxs used versus activity. All plots fitted well with the linear regression (the correlation coefficient was added in the figures). The points in parenthesis were omitted from the analyses. Data shown as mean ± sd (3 replicates). For the colour coding, see figure. Source data are provided as a Source Data file.

# Supplementary figure 6

**
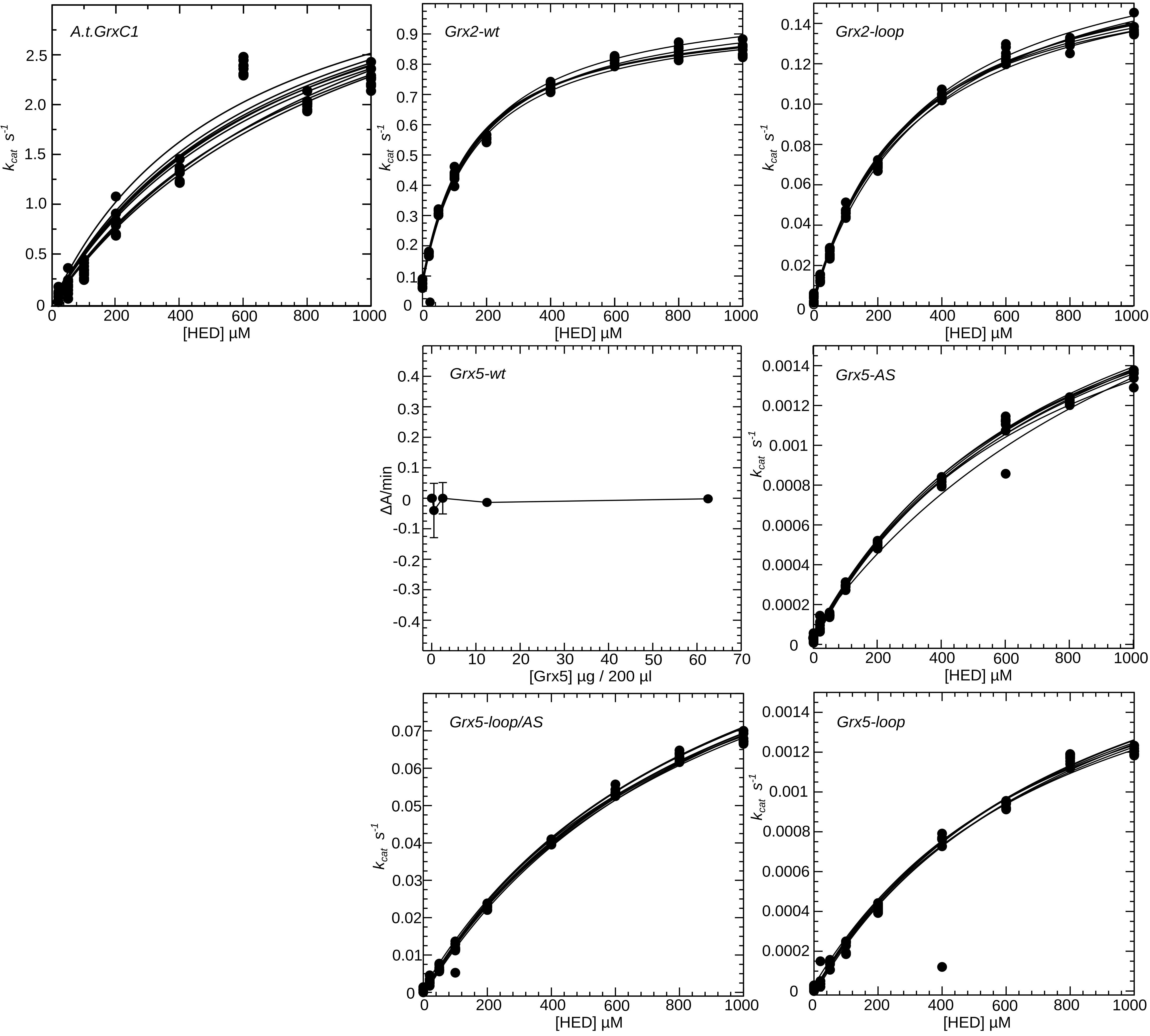
**

**Supplementary Figure 6 - Kinetic analysis of the CGFS-type and CxxC/S-type Grxs in the HED assay.** Michaelis-Menten plots of the HED concentration versus the catalytic activity. The curves represent the non-linear curve fitting to the original data (depicted as points) against the Michealis-Menten equation, n=7-8. Grx5-wt did not exhibit any activity in this assay as shown in the [enzyme] versus Δabsorbance-per-minute plot (mean ± sd, 8 biological replicates). Source data are provided as a Source Data file.

# Supplementary Figure 7

**
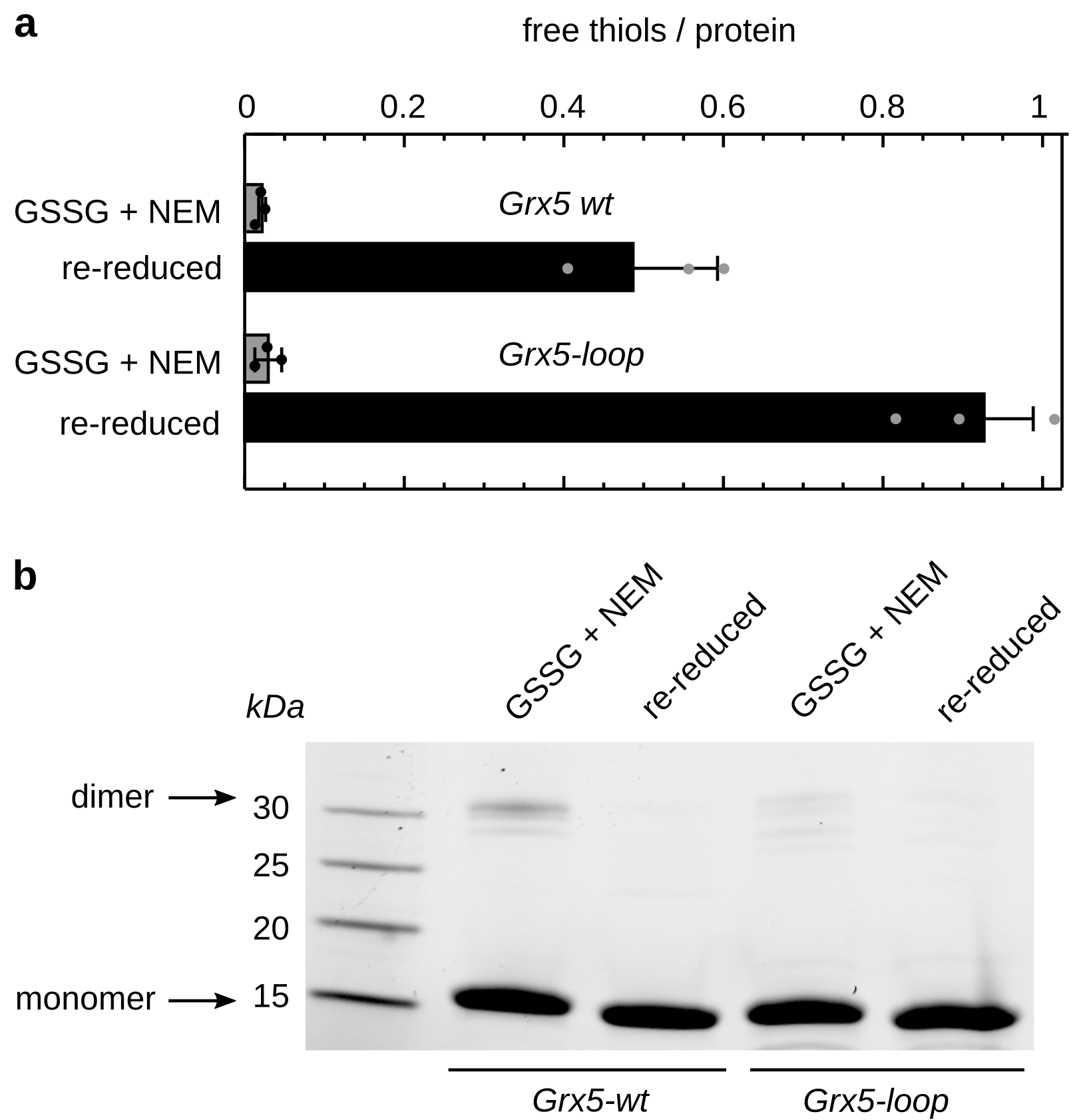
**

**Supplementary Figure 7 – Formation of the Grx-SG mixed disulfide.** The proteins were first reduced and re-buffered. The reduced proteins were oxidized with GSSG, all remaining free thiols were blocked by alkylation with NEM (sample: GSSG+NEM). Following re-buffering, the proteins were re-reduced with DTT (sample: re-reduced). **(a)** Quantification of free thiols in the sample using DTNB evinced the number of thiols/protein that formed the Grx-SG mixed disulfide **(b)** SDS PAGE of the samples as indicated. Grx5 contains, next to the active site cysteinyl residue one more cysteinyl residue (C122). To exclude that the formation of inter-molecular disulfides involving this residue accounted for the differences in free thiols, the samples were subjected to non-reducing and reducing SDS PAGE. Grx5-wt contained a minor fraction of disulfide bonded dimers, the Grx5-loop mutant did not form significant amounts of inter-molecular disulfides. The structurally unlikely formation of an intra-molecular disulfide between C62 and C122 would have yielded 2 free thiols, however, we never recorded more than one free thiol following alkylation and re-reduction. Data shown as mean ± sd, n=3 biological replicates). Source data are provided as a Source Data file.

# Supplementary figure 8


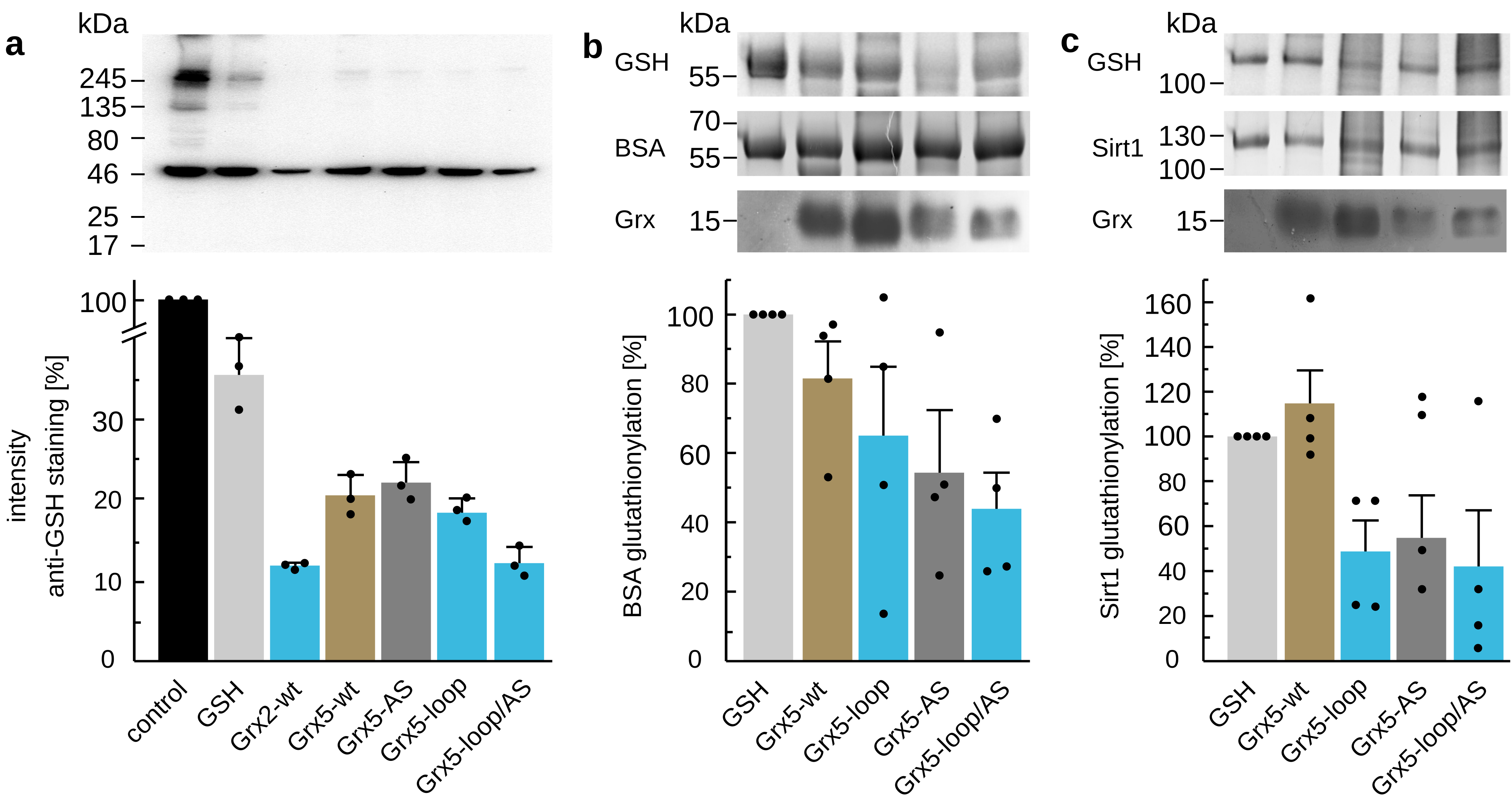


**Supplementary Figure 8 – Protein de-glutathionylation activity of different Grx mutants. (a)** 40 µg HeLa cell extract was incubated with 5 mM GSSG. After desalting, cell extracts were incubated with 1 mM GSH and ± 60 µM Grx mutants as indicated for 5 minutes. Proteins were separated by SDS-PAGE, glutathionylated proteins were visualized with anti-GSH antibodies after Western blotting and quantified (n=3, mean ± SD). (**b-c**) 10 µM of glutathionylated (Di-Eosin-GSSG) BSA **(b)** or Sirt1 **(c**) were incubated for 15 min with 1 mM GSH and ± 50 µM pre-reduced Grx mutants as indicated. After SDS-PAGE, fluorescent GSH was visualized by UV-light and proteins were stained with Coomassie Brilliant Blue. De-glutathionylation activity of Grx mutants were calculated correlating GSH signals with intensities of both substrates and Grxs (data shown as mean ± sd, n=3 biological replicates). Black bars represent the oxidized controls, light gray the reduction with GSH only, blue bars represents the proteins with the CxxC/S-type loop, brown bars proteins with the CGFS-type loop, the dark gray bar Grx5 with the CSYC active site of Grx2. Source data are provided as a Source Data file.

# Supplementary figure 9


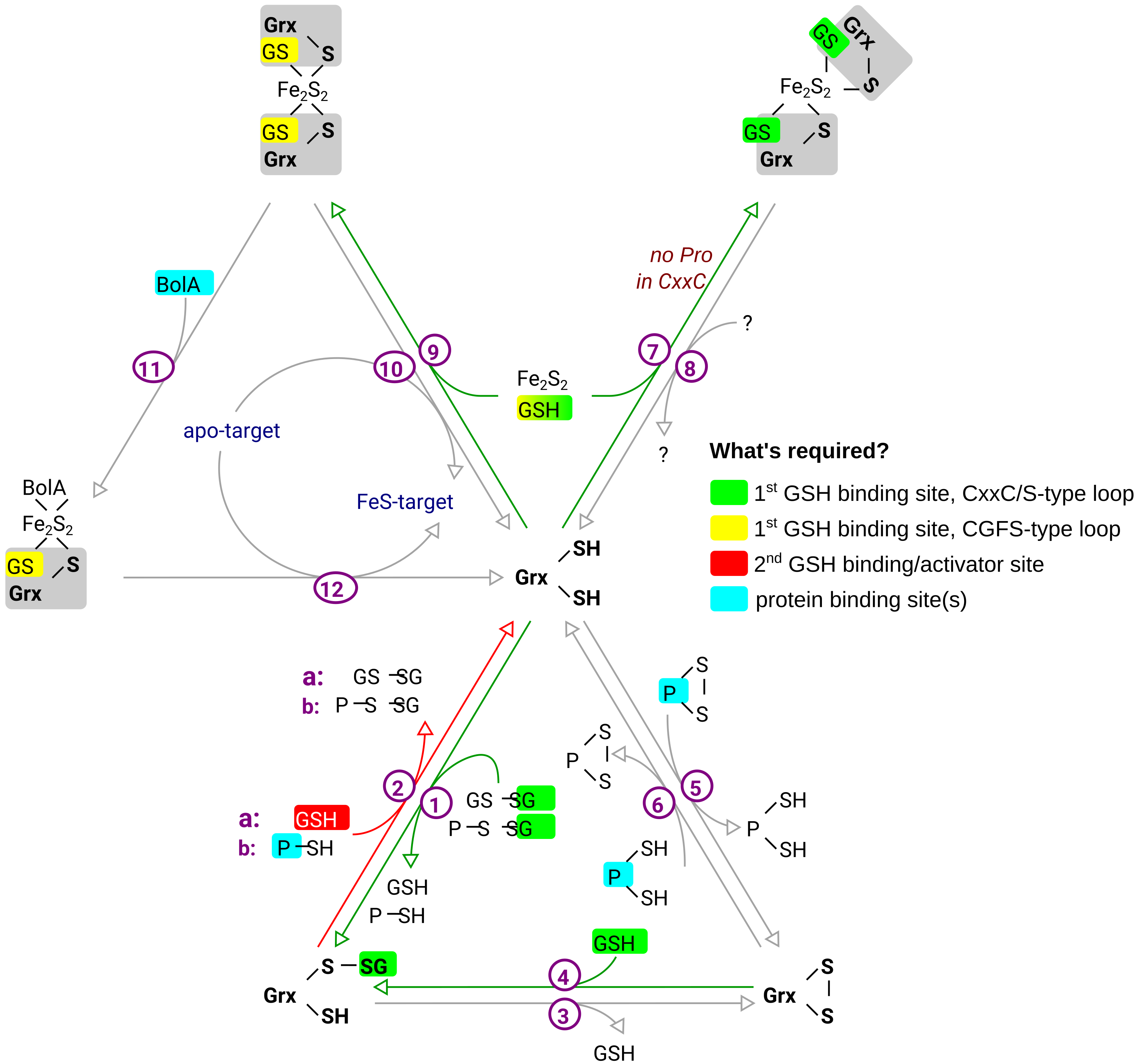
**Supplementary Figure 10 – Refined model of glutaredoxin reactions.** For the colour coding, see figure.

1. The reduction of a GSH-mixed disulfide (de-glutathionylation) or GSH disulfide requires the N-terminal active site thiol and the first GSH binding site with the preceding CxxC/S-type loop N-terminal of the active site (green). The product is a Grx-SG mixed disulfide.
2. This Grx-SG mixed disulfide can be reduced by a second GSH molecule requiring the second GSH binding site (red) or it can react with a protein thiol yielding a glutathionylated substrate. The latter requires a binding site for the respective target protein (blue).
3. Alternatively, some Grxs may also attack the GSH mixed disulfide with the second active site thiol to form an intramolecular Grx active site disulfide. This reaction competes with reaction 2^1^.
4. The reverse reaction requires the first GSH binding site with the CxxC/S-type loop preceding the active site (green) and is required for the reduction of Grxs, when these reduce protein disulfides in thiol-disulfide exchange reactions (dithiol mechanism, reaction 5).
5. Some Grxs can reduce protein disulfides directly in the dithiol reaction mechanism, i.e. in a thiol-disulfide exchange mechanism using both CxxC active site thiols. This reaction requires a binding site for the respective target protein(s). Examples for such reactions are the reduction of *E. coli* ribonucleotide reductase or phosphoadenylyl phosphosulfate reductase by Grxs^2,3^.
6. An example for the reverse reaction of (5) may be the reduction of the human Grx2 disulfide by thioredoxin reductase^1^.

Grxs lacking the prolyly residue at the second position of the active site motif can form an iron-sulfur cluster bridged dimeric holo complex^4–7^:

1. Such Grxs with the CxxC/S-type loop preceding the active site form a holo complex that may serve as redox sensor of the protein^8^.
2. The disassembly of the cluster, and thereby the activation of the proteins as oxidoreductase may be induced, for instance, be S-nitroso compounds^9^.
3. CGFS-typeGrxs with the longer loop preceding the active site bind GSH in a slightly different conformation (yellow), which has a profound influence on the location of the cluster and the relative orientation of the monomers towards each other in the holo complex^6,7^.
4. Clusters bound in this conformation may be directly transferred to target proteins^10,11^,
5. or via the formation of hetero-complexes with BolA-type proteins
6. be transferred to target proteins (see, e.g.,^12–14^).

# Supplementary references

1. Johansson, C., Lillig, C. H. & Holmgren, A. Human mitochondrial glutaredoxin reduces S-glutathionylated proteins with high affinity accepting electrons from either glutathione or thioredoxin reductase. *J. Biol. Chem.* **279**, 7537–7543 (2004).

2. Aslund, F., Ehn, B., Miranda-Vizuete, A., Pueyo, C. & Holmgren, A. Two additional glutaredoxins exist in Escherichia coli: glutaredoxin 3 is a hydrogen donor for ribonucleotide reductase in a thioredoxin/glutaredoxin 1 double mutant. *Proc. Natl. Acad. Sci. U. S. A.* **91**, 9813–9817 (1994).

3. Lillig, C. H. *et al.* New Thioredoxins and Glutaredoxins as Electron Donors of 3’-Phosphoadenylylsulfate Reductase. *J Biol Chem* **274**, 7695–7698 (1999).

4. Berndt, C. *et al.* How does iron-sulfur cluster coordination regulate the activity of human glutaredoxin 2? *Antioxid. Redox Signal.* **9**, 151–157 (2007).

5. Feng, Y. *et al.* Structural insight into poplar glutaredoxin C1 with a bridging iron-sulfur cluster at the active site. *Biochemistry* **45**, 7998–8008 (2006).

6. Johansson, C., Kavanagh, K. L., Gileadi, O. & Oppermann, U. Reversible sequestration of active site cysteines in a 2Fe-2S-bridged dimer provides a mechanism for glutaredoxin 2 regulation in human mitochondria. *J. Biol. Chem.* **282**, 3077–82 (2007).

7. Johansson, C. *et al.* The crystal structure of human GLRX5: iron-sulfur cluster co-ordination, tetrameric assembly and monomer activity. *Biochem. J.* **433**, 303–311 (2011).

8. Lillig, C. H. *et al.* Characterization of human glutaredoxin 2 as iron-sulfur protein: a possible role as redox sensor. *Proc. Natl. Acad. Sci. U. S. A.* **102**, 8168–8173 (2005).

9. Lepka, K. *et al.* Iron-sulfur glutaredoxin 2 protects oligodendrocytes against damage induced by nitric oxide release from activated microglia. *Glia* **65**, 1521–1534 (2017).

10. Bellí, G. *et al.* Structure-function analysis of yeast Grx5 monothiol glutaredoxin defines essential amino acids for the function of the protein. *J. Biol. Chem.* **277**, 37590–6 (2002).

11. Bandyopadhyay, S. *et al.* Chloroplast monothiol glutaredoxins as scaffold proteins for the assembly and delivery of [2Fe-2S] clusters. *EMBO J.* **27**, 1122–1133 (2008).

12. Li, H. & Outten, C. E. Monothiol CGFS Glutaredoxins and BolA-like Proteins: [2Fe-2S] Binding Partners in Iron Homeostasis. *Biochemistry* **51**, 4377–4389 (2012).

13. Couturier, J. *et al.* Monothiol glutaredoxin-BolA interactions: redox control of Arabidopsis thaliana BolA2 and SufE1. *Mol. Plant* **7**, 187–205 (2014).

14. Banci, L., Camponeschi, F., Ciofi-Baffoni, S. & Muzzioli, R. Elucidating the Molecular Function of Human BOLA2 in GRX3-Dependent Anamorsin Maturation Pathway. *J. Am. Chem. Soc.* **137**, 16133–16143 (2015).
