# Supplementary material for: Molecular basis for the distinct functions of redox-active and FeS-transfering glutaredoxins
Source: Nat Commun. 2020 Jul 10;11:3445. doi: 10.1038/s41467-020-17323-0 (PMC7351949; doi:10.1038/s41467-020-17323-0)
Supplement: Supplementary file 3 — Reporting Summary [file 41467_2020_17323_MOESM3_ESM.pdf]

nature research

Corresponding author(s): Christopher Horst Lillig

Last updated by author(s): Apr 20, 2020

Reporting Summary

Nature Research wishes to improve the reproducibility of the work that we publish. This form provides structure for consistency and transparency in reporting. For further information on Nature Research policies, see Authors & Referees and the Editorial Policy Checklist .

Statistics

For all statistical analyses, confirm that the following items are present in the figure legend, table legend, main text, or Methods section.

n/a	Confirmed	
☐	☒	The exact sample size (*n*) for each experimental group/condition, given as a discrete number and unit of measurement
☐	☒	A statement on whether measurements were taken from distinct samples or whether the same sample was measured repeatedly
☒	☐	The statistical test(s) used AND whether they are one- or two-sided<br>*Only common tests should be described solely by name; describe more complex techniques in the Methods section.*
☒	☐	A description of all covariates tested
☐	☒	A description of any assumptions or corrections, such as tests of normality and adjustment for multiple comparisons
☐	☒	A full description of the statistical parameters including central tendency (e.g. means) or other basic estimates (e.g. regression coefficient) AND variation (e.g. standard deviation) or associated estimates of uncertainty (e.g. confidence intervals)
☒	☐	For null hypothesis testing, the test statistic (e.g. *F*, *t*, *r*) with confidence intervals, effect sizes, degrees of freedom and *P* value noted<br>*Give P values as exact values whenever suitable.*
☒	☐	For Bayesian analysis, information on the choice of priors and Markov chain Monte Carlo settings
☒	☐	For hierarchical and complex designs, identification of the appropriate level for tests and full reporting of outcomes
☒	☐	Estimates of effect sizes (e.g. Cohen's *d*, Pearson's *r*), indicating how they were calculated

Our web collection on statistics for biologists contains articles on many of the points above.

Software and code

Policy information about availability of computer code

Data collection	Pymol v1.7.4, UCSF Chimera49 1.12, pdb2pqr 2.1.0, VMD 1.9.3, and APBS 1.5 were used for structural analysis.<br>MODELLER 9.24 and GROMACS 2019.3 were used for MD simulations<br>ImageLab 5.1 was used to obtain WB and gel images.<br>Clariostar 5.40 R3 (MARS 3.32) was used for kinetic measurments.
Data analysis	All numerical calculations (spectra, kinetics) were performed and visualized using grace-5.1.25 (https://plasma-gate.weizmann.ac.il/Grace/). Blot pictures were normalized using ImageLab 5.1 (Biorad) and Gimp 2.8.22 (https://www.gimp.org/). Densiometric analyses were performed using ImageLab 5.1 and ImageJ v1.51 (https://imagej.net). Structures were depicted using UCSF Chimera49 1.12. Picture panels and reaction schemes were generated using Inkscape 0.92 (https://inkscape.org/). MD simulations were run using MODELLER 9.24 (https://salilab.org/modeller/) and GROMACS 2016.3 (http://www.gromacs.org/). GSH complexes were prepared using ACPYPE (https://github.com/alanwilter/acpype). The constraint algorythm used was LINCS (https://doi.org/10.1021/ct700200b).

For manuscripts utilizing custom algorithms or software that are central to the research but not yet described in published literature, software must be made available to editors/reviewers. We strongly encourage code deposition in a community repository (e.g. GitHub). See the Nature Research guidelines for submitting code & software for further information.

Data

Policy information about availability of data

All manuscripts must include a data availability statement. This statement should provide the following information, where applicable:

- Accession codes, unique identifiers, or web links for publicly available datasets
- A list of figures that have associated raw data
- A description of any restrictions on data availability

Structures were aquired from the RSCB PDB Protein Data Bank (Http://www.rscb.org). Most of the data that support the findings of this study are available within the paper and its supplementary material. The data source file contains source data to the text, table 1, Figs. 2-6, suppl. table 2, and suppl. Figs. 2-8. Additional data

and materials will be made available by the corresponding author upon reasonable request.

Field-specific reporting

Please select the one below that is the best fit for your research. If you are not sure, read the appropriate sections before making your selection.

☒ Life sciences ☐ Behavioural & social sciences ☐ Ecological, evolutionary & environmental sciences

For a reference copy of the document with all sections, see nature.com/documents/nr-reporting-summary-flat.pdf

Life sciences study design

All studies must disclose on these points even when the disclosure is negative.

Sample size	We did not perform any statistical method to predetermine sample size. Based on our experience, 25 fish should be enough to see differences in a specific phenotype. For each experiment (survival and heme staining) we divided two standard fish clutches (approximately 200 eggs each) into the different injection conditions leading to a sufficient amount of fish to see relevant differences.
Data exclusions	In the kinetic anlysis, data points were excluded that were not within the linear range of the enzyme activity. The linear range was checked for each enzyme for both oxidation and reduction of roGFP2 (Suppl. Fig. 7 and 8). Values that fell of the linear range due to enzyme saturation or below the detection limit of the instrument were omitted.
Replication	All experiments were repeated independently 3 to 8 times. Reproducibility was confirmed by analysis of the mean +/- SD.
Randomization	In our zebrafish experiments injected eggs were randomly assigned to different groups. All samples other than zebrafish were allocated randomly.
Blinding	Blinding was not relevant to our study. Bias in the evaluation of numerical results was not expected. Evaluation of the binary (+/-) results of the zebrafish experiments is not affected by personal bias. Results were independently evaluated by 2 persons confirming each other.

Reporting for specific materials, systems and methods

We require information from authors about some types of materials, experimental systems and methods used in many studies. Here, indicate whether each material, system or method listed is relevant to your study. If you are not sure if a list item applies to your research, read the appropriate section before selecting a response.

Materials & experimental systems

n/a	Involved in the study	
☐	☒	Antibodies
☐	☒	Eukaryotic cell lines
☒	☐	Palaeontology
☐	☒	Animals and other organisms
☒	☐	Human research participants
☒	☐	Clinical data

Methods

n/a	Involved in the study	
☒	☐	ChIP-seq
☒	☐	Flow cytometry
☒	☐	MRI-based neuroimaging

Antibodies

Antibodies used	Abcam Anti-Glutathione antibody [D8] (ab19534), LOT: GR279251-32
Validation	Statement from the manufacturers homepage for the use in WB: 1/1000. Use under non reducing condition. We recommend blocking with 5% milk (not BSA). While glutathione itself is too small to detect in WB, this antibody will detect all glutathionylated proteins. You may observe multiple bands at variable molecular weights depending on what proteins in your samples are glutathionylated. (https://www.abcam.com/Glutathione-antibody-D8-ab19534.html; Tab "applications")

Eukaryotic cell lines

Policy information about cell lines

Cell line source(s)	HeLa cells, human , epithelial, cervix adenocarcinoma, originally obtained from Sten Orrenius, Karolinska Institutet Stockholm. We can not give information about the commercial source of the cells since Sten Orrenius passed away in April this year. His group cited the following source for the cells: doi:10.1084/jem.97.5.695.
Authentication	Phenotype

Mycoplasma contamination	Cell line is regularly tested for mycoplasm contamination via PCR. No contamination was detected.
Commonly misidentified lines (See ICLAC register)	No commonly misidentified cell lines were used in this study.

Animals and other organisms

Policy information about studies involving animals; ARRIVE guidelines recommended for reporting animal research

Laboratory animals	The zebrafish strain used in this study was Tuebingen long fin (http://zfin.org/ZDB-GENO-990623-2). Both female and male fish were used in this study. The zebrafish were analysed 24 or 48 hours post fertilisation.
Wild animals	No wild animals were used in this study.
Field-collected samples	No field collected samples were used in this study.
Ethics oversight	No ethical approval or guidance was required. According to the EU Directive 2010/63/EU on the protection of animals used for scientific purposes, early life-stages of zebrafish are not protected as animals until the stage of being capable of independent feeding (5 days post fertilization).

Note that full information on the approval of the study protocol must also be provided in the manuscript.
