# Supplementary material for: Molecular basis for the distinct functions of redox-active and FeS-transfering glutaredoxins
Source: Nat Commun. 2020 Jul 10;11:3445. doi: 10.1038/s41467-020-17323-0 (PMC7351949; doi:10.1038/s41467-020-17323-0)
Supplement: Supplementary file 2 — Peer Review [file 41467_2020_17323_MOESM2_ESM.pdf]

Reviewers' comments:

Reviewer #1 (Remarks to the Author):

This manuscript provides a systematic analysis of the structural differences between CxxC/S-type dithiol Grxs and CGFS-type monothiol Grxs in order to tease out the factors that explain their differing functions as oxidoreductases for the former, and Fe-S cluster chaperones for the latter. The authors provide convincing evidence that the specific active site residues and the length of the loop just a few residues before the active site are the two main factors that dictate the main function of the Grxs. These findings thus provide novel insight into the structure/function relationships of this important and ubiquitous class of proteins. Overall, the manuscript is clear, straightforward, and well-written and will be of broad interest to both the thiol redox and iron homeostasis research communities. However, there are a few issues that require clarification or additional data to support specific conclusions.

1. Supp Fig 3. Grx5-loop – why are the data points in red already at 30% oxidized at time 0? Why is there a lag in the rate of oxidation in the first 15 minutes in most samples? Is there a specific reagent added at this time point? Please explain. There is a similar 15-20 min lag in the data shown in Supp. Fig. 4.

2. Fig. 4a: Fe-S reconstitution: The authors should measure and report the Fe : S : protein mole ratios after reconstitution and desalting for each variant tested. These details will provide information about the efficiency of reconstitution. Was the Fe-S cluster occupancy similar for all samples? Furthermore, are the spectra in Fig 4A normalized to protein concentration or Fe-S cluster concentration? Please indicate in the figure caption for clarity.

3. Fig. 4b and Supp Fig. 7: These data would be more convincing if the experiments were repeated with three independent samples and the standard deviation calculated to evaluate the significance of the different decay rates. Additionally, the concentration of Fe-S-bound Grx used in the assays should be indicated in the figure captions or the methods section. Why is the Grx5-AS variant not shown in Fig. 4b (but is shown in Fig S7)? It would be better to have the data for this mutant from both assays to be consistent.

4. Fig. 5 caption: clarify the meaning of the symbols in a and c. Why is the rescue of heme biosynthesis so poor (~30%), even with Grx5-wt? The rescue quoted in the 2005 Nature paper (ref. 14 in the manuscript), which is similar to the method used in Fig. 5, was 95% when zebrafish were simultaneously injected with grx5 morpholino oligos and grx5 RNA. Please explain the discrepancy between the previous data and the data shown here.

5. Fig. 6C: These spectra would be easier to compare if the blue CD were overlaid in one panel and the brown CD overlaid in a panel below this. This would allow a better view of the relative intensities as well as the locations of the peaks and valleys in the CD spectra. Furthermore, the CD should also be normalized to [2Fe-2S] cluster concentration as done for the UV-visible absorption spectra to allow more accurate comparison of peak intensities. The concentration of Fe-S cluster in the sample should be indicated in the figure caption and the significance of the dotted lines clarified. The dotted lines would be easier to follow if one dot pattern or color corresponded to the Grx2 features and another dot pattern/color corresponded to the Grx5 features.

Reviewer limitations: I am not a structural or computational biochemist so I cannot carefully evaluate the methods used to obtain the results shown in Figs. 1 and 2.

Sincerely,
Caryn Outten

Reviewer #2 (Remarks to the Author):

Here, Trnka and colleagues investigated structural differences of class I and II glutaredoxins. The authors examined differences in loop structures and found that small distinctions between the two classes influence GSH binding and orientation, which determines whether a glutaredoxin will be redox active or able to bind Fe-S clusters. The study is designed well and laudable considering there is a resurgence in understanding the function of glutaredoxins and their involvement in redox signaling and Fe-S cluster biosynthesis.

My only major concern is associated with whether some of these mutants can either participate in Fe-S cluster biosynthesis or catalyze the deglutathionylation of proteins in cells. These mutants should be expressed in cultured cells (yeast or mammalian cell lines) and their capacity to either catalyze S-glutathionylation reactions or participate in Fe-S cluster biosynthesis should be assessed. Providing this information would add considerably to this study and strengthen the conclusions drawn by the authors.

A minor concern is related to the use of wild-type GrxC1 as a positive control. Using it as a control is fine but the data should be included and not listed as “not shown”.

Reviewer #3 (Remarks to the Author):

I have reviewed the manuscript titled “Molecular basis for the distinct functions of redox-active and FeS-transfering glutaredoxins” and it contains very interesting results on the qualitative side but fails to perform in quantitative aspects of the enzyme kinetics of their glutaredoxins. Briefly, the authors chose as assays a new one based on roGFP2 as detector of oxidation and reduction and the traditional HED assay. The roGFP2 assay is poorly described in terms of what it actually measures and while potentially interesting, needs a lot more work on the characterization of the reactions (1, 2 and 3 in the manuscript).

More in detail, the authors present in Table 1 the kinetic data of roGFP2 reduction and oxidation as “kcat” without explaining how those numbers were obtained, the SI shows time courses obtained under non-catalytic conditions (Grx is in some cases in excess over roGFP2) at only one concentration of substrate. With only these data it is impossible to assume that the enzyme would be saturated or even that the same reaction is seen under the different concentrations of Grx that go from 0.1 to 3.16 times the substrate concentration.

The authors indicate that the Grx - roGFP2 interaction was analyzed as described in ref 65. In fact ref 65 does not deal with enzymatic catalysis but only with interactions between roGFP2 and GSH/GSSG buffers with a few time courses and many endpoint/equilibrium measurements. One key piece of information missing in the kinetic analysis is what is the excitation spectrum of the intermediate roGFP2-S-SG? As this point is ignored both in the manuscript and in ref 65 two assumptions can be made:
A) The spectra of roGFP2-S-SG and roGFP2-(SH)2 are identical
B) The spectra of roGFP2-S-SG and roGFP2-(S-S) are identical

According to the reaction sequence presented (p. 7-8):
reaction 1. Grx-SH + GSSG ⇌ Grx-S-SG + GSH
reaction 2. Grx-S-SG + roGFP2-(SH)2 ⇌ Grx-SH + roGFP2-S-SG
reaction 3. roGFP2-S-SG ⇌ roGFP2-(S-S) + GSH
With assumption A, the fluorescence change appears only with reaction 3 and with assumption B only with reaction 2. Those discrepancies lead to the question: what is the rate limiting step under the

conditions studied? Is it the same under all conditions and Grx studied? If not, comparison of the results contained in table 1 is extremely difficult.
Let´s try with the example of Grx2 from Fig S3 first panel, with [Grx2] < [roGFP2] the slopes are slightly lower than the background reaction (!). Under stoichiometric conditions ([Grx2] = 1 μM) the plot indicates the oxidation of 0.6 μM roGFP2 in approximately 105 min (the 15 min lag time is unexplained) yielding an apparent rate constant of 0.6 μM /(105 min × 1 μM) = 5.7 × 10-3 min-1. An attempt to do the same with the 3.16 μM plot yields an apparent rate constant of 1.2 × 10-2 min-1 similar to the number presented in Table 1. Two comments on this analysis:
1) The apparent rate constants of 0, 0, 5.7 × 10-3 min-1 and 1.2 × 10-2 min-1 are different which precludes the assumption that only one catalytic reaction is observed.
2) The observed rate or slope of the time courses is not linear with [Grx2] and the reaction is only observed with [Grx2] ≥ [roGFP2].
And this is the example with the redox active enzyme.
My objection to the unfortunately “standard” HED assay is the following and does not really affect the qualitative conclusions of this manuscript, only its numerical results. In Fig S5 the x axis is [HED], while the KM results in Table 1 are referred to the actual substrate (βME-SG) as if both concentrations were equal. In the standard HED assay, HED is preincubated with an excess GSH (1 mM) for 3 minutes according to ref 33. The main problem is that the reaction between GSH and HED (reaction 4) is very slow and not quantitative. So, not all HED is transformed into βME-SG in equilibrium, much less in 3 minutes. The kinetic study of this reaction and many others involving low molecular weight thiols reacting non enzymatically has been in the literature for the past 40 years, but researchers keep making the same mistake of equating HED with βME-SG. For the record, the rate constant of GSH with HED, according to (Szajewski, R. P., and Whitesides, G. M. (1980) J. Am. Chem. Soc. 102, 2011–2026) is 12 M-1 min-1 at pH = 8, meaning that the reaction would have a t½ ≈ 57 min with 1 mM GSH and 0.2 mM HED. Also, upon equilibration (after several hours of reaction) the concentration of βME-SG would be approximately 0.11 mM.
The Grx assay involving βME-SG should not be used unless the substrate is available in pure form and its concentration is known.

Reviewer #1 (Remarks to the Author):

This manuscript provides a systematic analysis of the structural differences between CxxC/S-type dithiol Grxs and CGFS-type monothiol Grxs in order to tease out the factors that explain their differing functions as oxidoreductases for the former, and Fe-S cluster chaperones for the latter. The authors provide convincing evidence that the specific active site residues and the length of the loop just a few residues before the active site are the two main factors that dictate the main function of the Grxs. These findings thus provide novel insight into the structure/function relationships of this important and ubiquitous class of proteins. Overall, the manuscript is clear, straightforward, and well-written and will be of broad interest to both the thiol redox and iron homeostasis research communities. However, there are a few issues that require clarification or additional data to support specific conclusions.

1. Supp Fig 3. Grx5-loop – why are the data points in red already at 30% oxidized at time 0? Why is there a lag in the rate of oxidation in the first 15 minutes in most samples? Is there a specific reagent added at this time point? Please explain. There is a similar 15-20 min lag in the data shown in Supp. Fig. 4.

Response: The reaction was started by the addition of GSSG (oxidation) and GSH (reduction), respectively. The lag phase in the beginning was the phase before the addition of the starting reagents.

- We have clarified this in the captions to supplementary figures 3 and 4 (now 4 and 5).

We wish to thank reviewer 1 for pointing to the obvious pre-reaction that took place in some samples of the oxidation reaction, for instance the mentioned Grx5 loop mutant at higher concentrations. The pre-incubation mix contained already all reagents, except GSSG or GSH (see above). We were able to trace the problem down to partially oxidized Grxs in the pre-incubation mix that caused these background reactions. We have thus repeated the entire roGFP2 kinetics with fully reduced proteins to eliminate these problems. The results confirmed our original findings. We have changed the manuscript as follows:

- updated Table 1 with the data of the new kinetics
- updated figure 3 with the data of the new kinetics
- exchange of supplementary figure 3 (now 4) with the data of the new kinetics, now also including the data of our positive control (Arabidopsis thaliana GrxC1) and more clear colour coding
- exchange of supplementary figure 4 (now 5) now also including the data of our positive control (Arabidopsis thaliana GrxC1) and more clear colour coding

2. Fig. 4a: Fe-S reconstitution: The authors should measure and report the Fe : S : protein mole ratios after reconstitution and desalting for each variant tested. These details will provide information about the efficiency of reconstitution. Was the Fe-S cluster occupancy similar for all samples? Furthermore, are the spectra in Fig 4A normalized to protein concentration or Fe-S cluster concentration? Please indicate in the figure caption for clarity.

Response: We agree, and now included the determination of FeS content (from their absorptivity in UV-vis spectra) as well as the analysis of Fe by a colorimetric methods. Both FeS and Fe determination yielded very similar results. The efficiency of reconstitution was, for all proteins and mutants, always around 70% (now summarized in suppl. table 2). Hence, we did not observe differences in FeS occupancy between the different proteins following reconstitution.

- We have added this information to the result section:

  "To ensure similar FeS occupancy of all proteins, we reconstituted the Fe_2S_2 cluster in both wild-type and mutant Grx2 and Grx5, respectively. We have quantified the FeS content of the reconstituted proteins from molar absorptivity and by colorimetric methods. The results, summarized in suppl. table 2, demonstrate a similar FeS occupancy for all proteins at around 70%."

- We have added the experimental details to the Methods section:

  "Determination of iron content – The FeS content per Grx dimer was calculated from UV-vis spectra using the molar absorptivity of the cluster in 100% dimeric Grx2 at 430 nm as determined in ref.[68] ($\varepsilon430 = 3260\ M^{-1}\cdot cm^{-1}$) as reference. Protein bound iron was quantified as described previously[69] using 200 µM of in vitro freshly reconstituted proteins."

- We have added a new suppl. table 2 that summarizes the FeS and Fe determinations

The spectra in Fig. 4A were normalized to the molar absorptivity of the proteins (calculated using ProtParam from the ExPASy tool set, https://web.expasy.org/protparam/). Thus, the spectra also demonstrate the similarity in FeS occupancy between the proteins as they all show a similar absorptivity at, e.g. 430 nm following reconstitution.

- We have added this information to the caption of figure 4 as suggested:

  "The spectra were normalized to the molar absorptivity of the proteins calculated from their primary structures using ProtParam (https://web.expasy.org/protparam/)."

3. Fig. 4b and Supp Fig. 7: These data would be more convincing if the experiments were repeated with three independent samples and the standard deviation calculated to evaluate the significance of the different decay rates. Additionally, the concentration of Fe-S-bound Grx used in the assays should be indicated in the figure captions or the methods section. Why is the Grx5-AS variant not shown in Fig. 4b (but is shown in Fig S7)? It would be better to have the data for this mutant from both assays to be consistent.

Response: We agree and apologize for this carelessness. We have now included data for the cluster decay under ambient conditions at n = 3 for all Grx5 mutants. Moreover, we have analyzed the data in more detail and found that it fitted well with 1st order kinetics. We have also recalculated all the GSNO-induced cluster loss for all mutants and found that it fitted well with 2nd order kinetics. However, here it was our aim to analyze the influence of the distinct Grx structures on the binding of the FeS cluster itself and not on its reactivity with nitrosyl compounds. We have thus decided to remove these data from the manuscript.

The following changes were made:

- The method section was re-written as follows:
- *“Kinetics of FeS-cluster disassembly were followed at 420 nm at 25 °C. UV/Vis-spectra and kinetics were recorded by an UV-1800 spectrometer (Shimadzu Kyoto, Japan). FeS clusters were quantified as outlined below. All experiments were repeated independently at least three times. Initial cluster concentrations ranged from 80-250 µmol·l^{-1}. Rate constants were obtained from the slope of the integrated first order kinetics: $ln([FeS]_t \cdot [FeS]_{t=0}^{-1}) = k \cdot t$, where $[FeS]_t$ is the cluster concentration at time point t, $[FeS]_{t=0}$ the initial cluster concentration, k the first order rate constant and t the time in seconds.”*
- The result section was rephrased and the new data included:

  “We have also analyzed the stability of the Fe_2S_2 clusters under ambient conditions at 25°C. Clusters decay fitted well with first order kinetics (see the integrated first order kinetics in Fig. 4b). The clusters in Grx2-wt (first order rate constant: $(1.1 \pm 0.2)\cdot 10^{-5}$ s^{-1}) were more stable than the ones bound to Grx5-wt (k = $(4.1\pm0.6) \cdot 10^{-5}$ s^{-1}). This is in accordance with Grx5's function as FeS transferase. The Grx5-AS mutation stabilized the FeS clusters already (k = $(1.6\pm0.3) \cdot 10^{-5}$ s^{-1}) the loop and loop/As mutations in Grx5 stabilized the clusters to essentially the same rate constant as observed for Grx2: Grx5-loop k = $(9.4\pm1.7) \cdot 10^{-6}$ s^{-1}, Grx5-loop/AS k = $(1.2\pm0.2) \cdot 10^{-5}$ s^{-1}.”
- Figure 4b was exchanged and now depicts the linearized 1st order kinetics of the average of three independent experiments for the proteins analyzed.
- The legend to Fig. 4b was exchanged to:

  *“**(b)** Integrated first order kinetics of the decay of the FeS clusters of Grx2-wt, Grx5-wt, Grx5-AS, Grx5-loop, and Grx5-loop/AS under ambient conditions. The curves represent the average of three independent experiments. Initial cluster concentrations ranged from 50-150 µmol·l^{-1}. Rate constants were obtained from the slope of the curves following linear regression. $[FeS]_t$ is the cluster concentration at time point t, $[FeS]_{t=0}$ the initial cluster concentration.”*
- The former supplementary Fig. 7 and the text referring to it were removed.

4. Fig. 5 caption: clarify the meaning of the symbols in a and c. Why is the rescue of heme biosynthesis so poor (~30%), even with Grx5-wt? The rescue quoted in the 2005 Nature paper (ref. 14 in the manuscript), which is similar to the method used in Fig. 5, was 95% when zebrafish were simultaneously injected with grx5 morpholino oligos and grx5 RNA. Please explain the discrepancy between the previous data and the data shown here.

Response: We agree that our rescue efficiency is less than the efficiency published by Wingert et al. In their study, the rescue efficiency was already decreased when using Grx5 from yeast, mouse, and human instead of zebrafish Grx5. In our experiments, we additionally changed the mitochondrial transit sequence of both human Grx5 and human Grx2 to a transit sequence from *Neurospora crassa*, to guarantee the same efficiency of mitochondrial translocation of all proteins. This might have further decreased the rescue efficiency. Although rescue efficiency was not optimal, the

differences shown in nearly 800 individual fish clearly support the hypothesis of gain and loss of function of the engineered mutants.

- We have added the following section to the discussion section (3rd paragraph from bottom):
- *"Our in vivo results in the zebrafish (Fig. 5) confirm the gain of FeS cluster transfer function in the Grx2-loop mutant, even though our rescue efficiency was relatively low compared to the one published before[14]. Wingert et al. had already pointed out the decrease in rescue efficiency with Grxs from different species. In addition, we changed the mitochondrial transit sequence of all proteins to one from Neurospora crassa, to exclude effects caused by different mitochondrial translocation efficiencies. It is likely that this further decreased rescue efficiency. The differences demonstrated using nearly 800 individual fish, however, support our conclusion that the loop is the major determinant discriminating between oxidoreductase activity and FeS transferase activity."*
- We have amended the caption to figure 5 as suggested:

  "… The arrow with cross in (a) points to a dead embryo. … The arrows in (c) point to heme-positive (+) and negative (-) embryos. …"

5. Fig. 6C: These spectra would be easier to compare if the blue CD were overlaid in one panel and the brown CD overlaid in a panel below this. This would allow a better view of the relative intensities as well as the locations of the peaks and valleys in the CD spectra. Furthermore, the CD should also be normalized to [2Fe-2S] cluster concentration as done for the UV-visible absorption spectra to allow more accurate comparison of peak intensities. The concentration of Fe-S cluster in the sample should be indicated in the figure caption and the significance of the dotted lines clarified. The dotted lines would be easier to follow if one dot pattern or color corresponded to the Grx2 features and another dot pattern/color corresponded to the Grx5 features.

Response: We have carefully evaluated several different options to improve Fig. 6C, including the suggested overlay. However, we found that the individual spectral properties, similarities, and differences become too obfuscating when overlayed, even when scaled up in y-direction. We have thus, as suggested, adopted the brown/blue coloring scheme also for the dotted lines highlighting the discussed features. In addition, we have included arrow heads and the respective wavelength at the different points of reference. We are convinced that this allows best to explain and depict the similarities and differences between the individual spectra.

The spectra were not normalized, because all proteins were analysed at identical FeS concentration that was calculated from the proteins' absorptivity at 430 nm. Hence, the spectra can be compared for the ellipticity features and intensities directly. We have, as suggested, included the details on FeS concentration in the figure caption.

The manuscript was changed as follows:

- Fig. 6C was changed as outlined above.
- The description in the result section was amended as follows (grey background, last paragraph of the result section):

"Our functional analysis of the proteins suggested that the Grx2-loop and the Grx5-loop mutants should form holo-complexes that reflect the conformations of the other Grx class. As depicted in Fig. 6c, the Grx5-loop mutant shows essentially the same features as Grx2-wt. In particular, it lost the Grx5-specific maximum at 362 nm and minimum at 408 nm. The Grx2-loop mutant displays features that better reflect the characteristics of the Grx5-wt protein than those of its Grx2-wt parent protein, it gained the Grx5-specific maximum in the region of 362 (shifted to 370) nm the minimum in the region of 408 nm (shifted to 420, Fig. 6c). The exchange of the active site in Grx5 resulted in CD properties that lay in between those of the two wild-type proteins."

- The following sentence was included in the Methods section describing CD spectroscopy:

  "CD spectra were recorded […] with the FeS holo-proteins at 175 µM concentration. […] The concentrations of the holo proteins were determined based on the molar absorptivity of the FeS-holo proteins at 430 nm (ε_{430} = 3260 $M^{-1}\cdot cm^{-1}$)[68]. […]"

- The legend to Fig. 6 was completed in the flowing way:

  "[…] The concentration of the FeS holo-proteins was adjusted to 175 µM using the molar absorptivity of the FeS clusters for all CD spectra recorded."

Reviewer #2 (Remarks to the Author):

Here, Trnka and colleagues investigated structural differences of class I and II glutaredoxins. The authors examined differences in loop structures and found that small distinctions between the two classes influence GSH binding and orientation, which determines whether a glutaredoxin will be redox active or able to bind Fe-S clusters. The study is designed well and laudable considering there is a resurgence in understanding the function of glutaredoxins and their involvement in redox signaling and Fe-S cluster biosynthesis.

My only major concern is associated with whether some of these mutants can either participate in Fe-S cluster biosynthesis or catalyze the deglutathionylation of proteins in cells. These mutants should be expressed in cultured cells (yeast or mammalian cell lines) and their capacity to either catalyze S-glutathionylation reactions or participate in Fe-S cluster biosynthesis should be assessed. Providing this information would add considerably to this study and strengthen the conclusions drawn by the authors.

Response: Our study aimed to identify the factors that separate FeS-transferring Grxs from redox-active Grxs. We agree, that it is of highest importance to provide strong evidence for our hypothesis that the different loop structures preceding the active site are in fact the crucial elements.

Regarding the analysis of FeS transfer *in vivo*, we demonstrate in our complementation study in zebrafish, i.e. whole organisms, that our engineered mutants of the redox-active Grx2 become able to complement the lethal effect of the silencing of the FeS-transfering Grx5. Since the legality of this phenotype depends on the FeS transferring activity of Grx5 (Wingert et al. from our reference

list), we feel safe to conclude that the engineered Grx2-loop mutant can facilitate this reaction *in vivo*, i.e. in an intact organism.

Regarding the ability of the engineered Grx5 mutants to catalyze the reversible (de-)glutathionylation of proteins, the roGFP2 oxidation and reduction both depend on the reversible glutathionylation of the roGFP2 substrate by the Grxs (for details, see our response to reviewer 3's comments). It thus already demonstrates the ability of the proteins to catalyze this reaction. To further strengthen this conclusion, we have now included three more experiments demonstrating this activity: The reduction of glutathionylated proteins in HeLa cell extracts and of the purified proteins Sirt1 and BSA, both of which can be detected glutathionylated under physiological or pathological conditions. These data are summarized in a new supplementary figure (new suppl. Fig. 8). In summary, these results confirm the ability of Grx2 and the engineered Grx5 mutants to facilitate this reaction with different protein substrates.

The following changes were made in the manuscript:

- The following paragraph was added to the result section:

  "The oxidation and reduction of roGFP2 requires the formation of an intermediate protein-glutahione mixed disulfide (see above). To analyze the ability of the Grxs to facilitate the reversible (de)-glutathionylation of other proteins as well, we have analyzed their ability to de-glutathionylate proteins in HeLa cell extracts, and purified BSA and Sirt1 (supplementary Fig. 8). In all cases, the Grx5 double mutant Grx5-loop/AS de-glutathionylated the proteins most efficiently, while – with the exception of Sirt1 – both the Grx5-loop and Grx5-AS mutants were less efficient. Wild-type Grx5 showed low (HeLa extract and BSA) or no (Sirt1) activity."

- The discussion section on this topic was modified in the following way (grey background):

  […] Both the reduction of roGFP2 as well as the HED assay require the reaction of the Grx-S-SG intermediate with a second molecule of GSH (reaction 1 reverse and reaction 6, see above), this second GSH binding site has only been established recently[10]. The additional exchange of the CGFS to the CSYC active site further increased the activity of the loop mutant. This was also true for the de-glutathionylation of the other proteins analyzed, the Grx5 double mutant was always the most efficient. Our results suggest that the Gly-Phe residues prefer a conformation that hinders the interaction with the second GSH molecule, likely by blocking or shifting the access to the mixed Grx-S-SG disulfide that has to be attacked in a 180° angle to form the tertiary intermediate required for the thiol-disulfide exchange reaction[28]. […].

- The experimental procedures were completed with the following details:

  "Analysis of protein de-glutathionylation – De-glutathionylation using single substrates follows a protocol published in[65]. 30 µM of BSA (Sigma) or recombinant D. rerio Sirt1 were reduced with 10 mM DTT, desalted (Zeba spin, Thermo Fisher), glutathionylated with 150 µM Di-Eosin-GSSG (kind gift of Arne Holmgren), and desalted again. 10 µM substrate were incubated for 15 min at 37 °C with 1 mM GSH and ± 50 µM pre-reduced Grx mutants. After SDS-PAGE, fluorescent GSH was visualized by UV-light and proteins were stained

with Coomassie. For the de-glutathionylation of whole lysates, 40 µg HeLa cell extract was incubated with 5 mM GSSG. After desalting (PD10, GE Healthcare), cell extracts were incubated with 1 mM GSH and ± 60 µM Grx mutants for five minutes. Proteins were separated by SDS-PAGE, glutathionylated proteins were visualized with anti-GSH antibodies (#ab19534, abcam) after Western blotting."

- The new supplementary Figure 8 was included.

A minor concern is related to the use of wild-type GrxC1 as a positive control. Using it as a control is fine but the data should be included and not listed as "not shown".

Response: We agree and have now included full kinetics for the roGFP2 assays also for our classical CxxC-type GrxC1 positive control from A. thaliana. In addition, see our response to reviewer 1: we have repeated the full roGFP2 kinetics and modified the main text accordingly. The following changes were made:

- Suppl. Fig. 3, 4, and 5(now 4-6) and the respective captions were complemented with the kinetics of A.t. GrxC1.
- The data were included in table 1 in the main text.
- The following changes were made to the result section of the main text (grey background):

  "Grx2 catalyzed the reaction at reasonable rates, comparable to those of the classical CxxC-type Arabidopsis thaliana GrxC1 that was used as a highly efficient positive control in all reactions (table 1, supplementary Fig. 4-5). Introduction of the alternative loop in the Grx2-loop mutant led to a loss of 78% of its activity in the oxidative reaction. This reaction was also the only one in which wild-type Grx5 (Grx5-wt) shows some activity, approx. 42% of the activity of Grx2. Exchange of the active site to those of Grx2 increased the activity to 214%. The introduction of the CxxC/S-type loop increased the activity further to 285%, the combined Grx5-loop/AS mutation to 337% (Fig. 3a, details in suppl. Fig. 4). Clearly, the shortening of the loop alone was sufficient to turn Grx5 into a highly efficient catalyst of roGFP2 oxidation.
  Reduction of oxidized roGFP2 takes place in reaction order 3-2-1 (see above). The extension of the loop in the Grx2-loop mutant decreased the rate to 0.5%. Grx5-wt is inactive in this reaction, exchange of the active site (Grx5-AS) yielded an activity of approx 1% of Grx2-wt. The CxxC/S-type loop in the Grx5-loop mutant increased the rate to 1.7% compared to Grx2-wt with some additional additive effect of the active site swap (table 1, Fig. 3b, suppl. Fig. 5). […]"

Reviewer #3 (Remarks to the Author):

I have reviewed the manuscript titled "Molecular basis for the distinct functions of redox-active and FeS-transfering glutaredoxins" and it contains very interesting results on the qualitative side but fails to perform in quantitative aspects of the enzyme kinetics of their glutaredoxins. Briefly, the

authors chose as assays a new one based on roGFP2 as detector of oxidation and reduction and the traditional HED assay. The roGFP2 assay is poorly described in terms of what it actually measures and while potentially interesting, needs a lot more work on the characterization of the reactions (1, 2 and 3 in the manuscript).

More in detail, the authors present in Table 1 the kinetic data of roGFP2 reduction and oxidation as "kcat" without explaining how those numbers were obtained, the SI shows time courses obtained under non-catalytic conditions (Grx is in some cases in excess over roGFP2) at only one concentration of substrate. With only these data it is impossible to assume that the enzyme would be saturated or even that the same reaction is seen under the different concentrations of Grx that go from 0.1 to 3.16 times the substrate concentration.

Response: We agree with reviewer 3 to the most. Regarding the non-catalytic conditions with Grxs: The concentrations of the Grxs are in fact in some cases in access over those of the roGFP2 concentration. As depicted in all our kinetic pictures, however, the reaction of roGFP2 (reduced) with GSSG and roGFP2 (oxidized) with GSH are very slow (see the grey curves in suppl. Fig 4 and 5). The addition of the Grxs, although at higher concentration, dramatically increases the velocity of this reaction. This is the pure definition of catalysis: increased reaction velocity without changing the thermodynamic equilibrium of the reaction. This has been well documented before, see for instance ref 65.

We do, of course, agree with reviewer 3 that under these conditions we operate outside the classical Michaelis-Menten conditions. This is also the reason, why we never attempted to obtain a Michaelis constant. Under Michaelis-Menten conditions, the mass balance $[E]_{total} = [E] + [ES]$ is the major objective, while the formation of [ES] complex would essentially not change the total substrate concentration $[S]_{total}$. Under the condition of our roGFP2 assay here (and likely under many physiological conditions), when the [E] is in the same order of magnitude as [S], a second mass balance has to be taken into account: $[S]_{total} = [S]_{free} + [ES]$ In consequence, substrate depletion by formation of enzyme-substrate complexes occurs, and substrate saturation of the enzyme, i.e. $[E]_{total} = [ES]$, cannot be achieved. Test conditions are limited by the fluorescence intensities that can be recorded, the upper limit being around 1.6 µM roGFP2 when the detector of the instrument becomes saturated. It is thus not possible to obtain further data from Dixon plots or likewise since this would require knowledge of V_{max}.

We have thus decided to stick to a single roGFP2 concentration close to the saturation limit of fluorescence detection, i.e. 1 $\mu mol \cdot l^{-1}$. The apparent turn over number we calculated was the initial rate of the reaction under these conditions (derived from the 1st derivatives of the original plots). This apparent turn over number allows a comparison between the functionalities and to some degree efficiencies of different redoxins, however, without discriminating between affinity towards the substrate and maximal turn over. Since the major aim of our study was to analyze the functionalities of the proteins and engineered mutants we have employed this assay. Most notably this new assay offers the unique opportunity to study the reduction and oxidation of the same substrate in continuous assays. We have added the following notes to the manuscript:

- To the main text (methods section): "[…] *The k_{cat} calculated was defined as the specific initial rate of roGFP2 oxidation and reduction, respectively, at 1$\mu mol \cdot l^{-1}$ substrate concentration.*"

- And to the legend of Fig. 3: "[…]; k_{cat} *here was defined as the specific initial rate of roGFP oxidation and reduction, respectively, at 1µmol·l*$^{-1}$ *substrate concentration.* […]"

The authors indicate that the Grx - roGFP2 interaction was analyzed as described in ref 65. In fact ref 65 does not deal with enzymatic catalysis but only with interactions between roGFP2 and GSH/GSSG buffers with a few time courses and many endpoint/equilibrium measurements. One key piece of information missing in the kinetic analysis is what is the excitation spectrum of the intermediate roGFP2-S-SG? As this point is ignored both in the manuscript and in ref 65 two assumptions can be made:

A) The spectra of roGFP2-S-SG and roGFP2-(SH)2 are identical

B) The spectra of roGFP2-S-SG and roGFP2-(S-S) are identical

According to the reaction sequence presented (p. 7-8):

reaction 1. Grx-SH + GSSG ⇌ Grx-S-SG + GSH

reaction 2. Grx-S-SG + roGFP2-(SH)2 ⇌ Grx-SH + roGFP2-S-SG

reaction 3. roGFP2-S-SG ⇌ roGFP2-(S-S) + GSH

With assumption A, the fluorescence change appears only with reaction 3 and with assumption B only with reaction 2. Those discrepancies lead to the question: what is the rate limiting step under the conditions studied? Is it the same under all conditions and Grx studied? If not, comparison of the results contained in table 1 is extremely difficult.

Let´s try with the example of Grx2 from Fig S3 first panel, with [Grx2] < [roGFP2] the slopes are slightly lower than the background reaction (!). Under stoichiometric conditions ([Grx2] = 1 µM) the plot indicates the oxidation of 0.6 µM roGFP2 in approximately 105 min (the 15 min lag time is unexplained) yielding an apparent rate constant of 0.6 µM /(105 min × 1 µM) = 5.7 × 10-3 min-1. An attempt to do the same with the 3.16 µM plot yields an apparent rate constant of 1.2 × 10-2 min-1 similar to the number presented in Table 1. Two comments on this analysis:

1) The apparent rate constants of 0, 0, 5.7 × 10-3 min-1 and 1.2 × 10-2 min-1 are different which precludes the assumption that only one catalytic reaction is observed.

2) The observed rate or slope of the time courses is not linear with [Grx2] and the reaction is only observed with [Grx2] ≥ [roGFP2].

And this is the example with the redox active enzyme.

Response: We agree with reviewer 3 and have now added the missing characterization in form of a new Suppl. Fig. 3. Here, we have used a roGFP2 mutant lacking the second cysteinyl residue required for formation of the disulfide to trap the glutathionylated intermediate. The results demonstrate that:

1. The ratiometric change in excitation properties does not occur in the absence of the second cysteinyl residue (suppl. Fig. 3a-d).
2. The hypothesized glutathionylated intermediate can be detected by mass spectrometry (suppl. Fig. 3e-f)

3. The roGFP-SG intermediate displays exactly the same spectral properties as the reduced protein (suppl. Fig. 3d).
4. The oxidation of roGFP2 to the intra-molecular disulfide occurs fast and when the reaction mixture of this 2-Cys roGFP2 protein was analyzed by mass spectrometry, we could not detect the glutathionylated roGFP2 intermediate.

The reaction of reduced Grxs with GSSG is expected to be fast, Rabenstein and Millis (Biochim. Biophys. Acta 1249:29-30, 1995) recorded a second order rate constant of $7 \cdot 10^5$ for Grx1 from pigs, for instance. We are thus confident to conclude that the rate limiting step for the oxidation of the model substrate roGFP2 is the formation of the roGFP2-SG intermediate (reaction 2).

As outlined in our response to reviewer 1, we have re-run the full roGFP2 kinetic analysis due to a background reaction caused by partially oxidized Grxs in the pre-incubation mix of our assay. We have now also included plots on the linearity of the [E] versus turn-over number for the proteins analyzed in suppl. Fig. 4 and 5 (formerly 3 and 4). In fact, the average turn over numbers were calculated from these data (table 1). With the exception of the highest concentration (3.16 µM) for the most active Grxs (that expectedly displayed a lower specific activity) and the lowest for the least active (that could not be distinguished from background) we obtained good correlations, i.e. a linear dependence of the turn-over numbers on the concentrations of the Grxs. We included the correlations coefficients of the linear regressions of these plots now also in suppl. Figs. 4 and 5.

The manuscript was changed in the following way:

- Table 1 was updated with the data from the repeated analyses. These confirm our previous observations.
- The lag phase in the beginning of the reactions was explained (pre-incubation before the addition of GSSG and GSH, respectively, to start the reactions). We also included an arrow in all plots of suppl. Figs 4 and 5 (formerly 3 and 4) to indicate theses time points.
- [Grx] versus reaction velocity plots were included in suppl. Fig. 4 and 5 (formerly 3 and 4) including linear regression analyses, all of which demonstrate linear dependence of the reaction on [Grx].
- The following paragraph was added to the result section:

  "[reactions 1-3] ... We have confirmed this reaction sequence using mass spectrometry and a roGFP2 mutant lacking the second cysteinyl residue required for reaction 2 to trap the glutathionylated roGFP2 intermediate (summarized in suppl. Fig. 3). Notably, this roGFP2-SG intermediate is spectroscopically indistinguishable from the reduced protein. The kinetics of fluorescence changes must therefore reflect the overall reaction. In the reaction of the Grx-catalyzed oxidation of the 2-Cys roGFP2 by GSSG no significant amounts of the roGFP2-SG intermediate could be detected. We feel thus confident to conclude that oxidation of roGFP2 through Grxs is facilitated in steps 1-2-3; reaction 2 is rate limiting for the full oxidation of roGFP2 and the ratiometric change in fluorescence excitation."

My objection to the unfortunately "standard" HED assay is the following and does not really affect the qualitative conclusions of this manuscript, only its numerical results. In Fig S5 the x axis is [HED], while the KM results in Table 1 are referred to the actual substrate (βME-SG) as if both concentrations were equal. In the standard HED assay, HED is preincubated with an excess GSH (1 mM) for 3 minutes according to ref 33. The main problem is that the reaction between GSH and HED (reaction 4) is very slow and not quantitative. So, not all HED is transformed into βME-SG in equilibrium, much less in 3 minutes. The kinetic study of this reaction and many others involving low molecular weight thiols reacting non enzymatically has been in the literature for the past 40 years, but researchers keep making the same mistake of equating HED with βME-SG. For the record, the rate constant of GSH with HED, according to (Szajewski, R. P., and Whitesides, G. M. (1980) J. Am. Chem. Soc. 102, 2011–2026) is 12 M-1 min-1 at pH = 8, meaning that the reaction would have a $t_{½} \approx 57$ min with 1 mM GSH and 0.2 mM HED. Also, upon equilibration (after several hours of reaction) the concentration of βME-SG would be approximately 0.11 mM.

The Grx assay involving βME-SG should not be used unless the substrate is available in pure form and its concentration is known.

Response: We agree with reviewer 3 that this assay is numerically difficult to analyze. There is, however, a huge body of literature with kinetic data describing various glutaredoxins from all kingdoms of life. In fact, we had been prompted by several colleagues to include this assay for comparability with previous reports. We are convinced that this data is valuable for comparisons and (as also noted by reviewer 3) does not affect our conclusions but rather strengthen and complete them.

- We have, as requested implicitly, exchanged the K_m (bME-SG) for $K_{m\,app}$ (HED).
- In addition, the following note were added to the manuscript (result section):

  "[reactions 4-6] The HED assay is numerically not exact, since [HED] in the assay does not correspond to the [βME] in a 1:1 stoichiometric manner [30]. The assay, does however allow to compare apparent catalytic efficiencies. [...]"

REVIEWERS' COMMENTS:

Reviewer #1 (Remarks to the Author):

The authors have effectively addressed all of the reviewers' concerns. However, there is a typo in Fig. 4b. Presumably, the solid blue line is Grx2-WT, not Grx5-WT.

Reviewer #2 (Remarks to the Author):

The authors have a satisfactory response to the concerns raised.

REVIEWERS' COMMENTS:

Reviewer #1 (Remarks to the Author):

The authors have effectively addressed all of the reviewers' concerns. However, there is a typo in Fig. 4b. Presumably, the solid blue line is Grx2-WT, not Grx5-WT.

Response: We apologize for this error. It was corrected in the revised manuscript.

Reviewer #2 (Remarks to the Author):

The authors have a satisfactory response to the concerns raised.
